# A platform for efficient, thiol-stable conjugation to albumin's native single accessible cysteine†
†Electronic supplementary information (ESI) available: LC-MS, ES-MS and deconvoluted spectra for all reactions with proteins described herein, and ^1^H and ^13^C NMR spectra for all small molecule constructs. See DOI: 10.1039/c5ob01205h



**DOI:** 10.1039/c5ob01205h

**Published:** 2015-06-25

**Authors:** Mark E. B. Smith, Mikael B. Caspersen, Eifion Robinson, Maurício Morais, Antoine Maruani, João P. M. Nunes, Karl Nicholls, Malcolm J. Saxton, Stephen Caddick, James R. Baker, Vijay Chudasama

**Affiliations:** a Department of Chemistry , University College London , London , WC1H 0AJ , UK . Email: j.r.baker@ucl.ac.uk ; Email: v.chudasama@ucl.ac.uk ; Tel: +44 (0)20 7679 2653, +44 (0)20 7679 2077; b Novozymes Biopharma UK Ltd , Castle Court , 59 Castle Boulevard , Nottingham , NG7 1FD , UK

## Abstract

Herein we report the use of bromomaleimides for the construction of stable albumin conjugates *via* conjugation to its native, single accessible, cysteine followed by hydrolysis. Advantages over the classical maleimide approach are highlighted in terms of quantitative hydrolysis and absence of undesirable retro-Michael deconjugation.

## Abstract 2

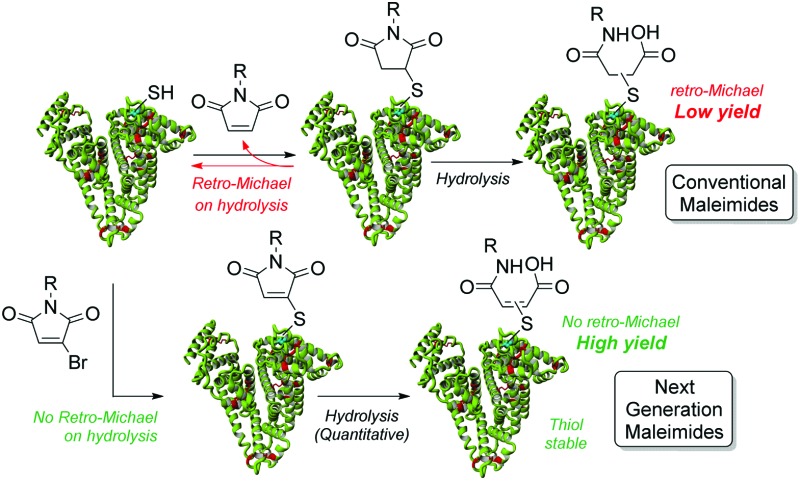
Thiol-stable albumin biologics are enabled by controlled, quantitative hydrolysis of maleimide–albumin conjugates, *i.e.* with no retro-Michael.

The serum half-life of a drug can be increased by conjugation to various entities.^1^ In general, strategies operate by increasing the size of the overall construct to minimise renal clearance or through enabling recycling *via* the neonatal Fc receptor (FcRn).^1^ Thus, human serum albumin is an excellent conjugation candidate for serum half-life extension as it offers both of these features (*t*_1/2_ albumin ≈ 19 days).^2^ Accordingly the use of albumin for drug delivery has been proven in the clinic and GSK has launched Eperzan® (2014), which is an albumin-GLP-1 fusion for the treatment of type 2 diabetes mellitus in adults.^2,3^

Owing to the favourable properties of albumin, various strategies have been employed to extend the circulatory half-life of numerous entities by engaging them covalently or transiently with this protein.^2^ As albumin has a single free thiol (cysteine 34) available for conjugation, covalent conjugation *via* reaction at this position has proved to be a very popular strategy for attachment.^2^ This strategy has been used to extend the half-life of various protein-based drugs, including granulocyte colony stimulating factor (G-CSF),^4^ Kringle domain,^5^ DARPin domain,^6^ the antiretroviral gp41 targeting peptide C34 (PC-1505),^7^ insulin,^8^ the opioid agonist dynorphin A (CJC-1008),^9^ YY peptide^10^ and GLP-1/exendin-4 (CJC-1131 and CJC-1134-PC).^11^ Lysine modification strategies have also been trialled,^2^ however, these approaches result in hetereogeneous mixtures (due to a large number of surface accessible lysines on albumin), limit solubility (by removal of charged groups) and may result in denaturing.^12^

Cysteine 34 is located close to the surface of the albumin protein in a shallow crevice (Fig. 1). It is situated in a rather anionic environment and has relatively limited solvent accessibility.^2,13^ This environment infers some unique properties on the thiol, and it has a p*K*_a_ of approximately 8.5 in the absence of external factors *in vivo*.^13^

**Fig. 1:**
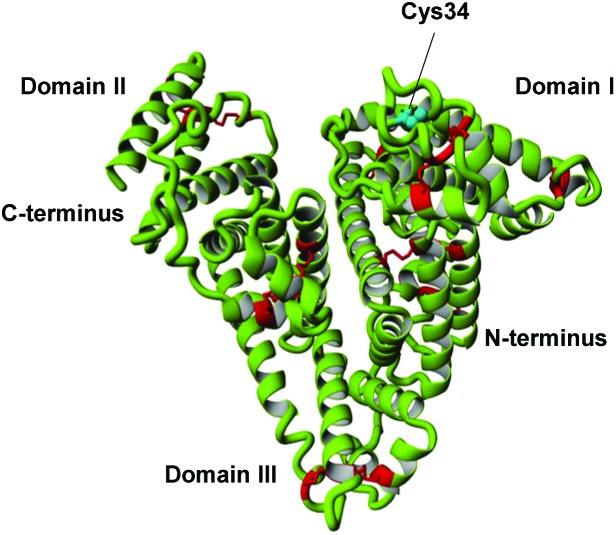
Structure of albumin, highlighting cysteine 34 and key features.

Historically, for conjugation to a free thiol on cysteine, maleimide chemistry has been used. Thus, it is no surprise that maleimides have been shown to react with the thiolate of cysteine 34 of albumin **1** in an efficient manner to form succinimide-albumin conjugates, **2** (Scheme 1).^2,4–11^ A pH of 7.4 is generally used to ensure that there is enough of the thiolate available whilst minimizing deprotonation of ammonium groups, *i.e.* to perturb side-reactions of the protein amino groups with maleimide. However, it has recently come to light that the thioether bond on the resultant succinimide is not robust.^14^ The succinimide can revert back to maleimide and free thiol *via* a retro-Michael pathway. Thus, highly undesirably, the released maleimide may react with other thiol reactive species and the released thiol may react with other compounds *in vivo*.^14^

**Scheme 1:**
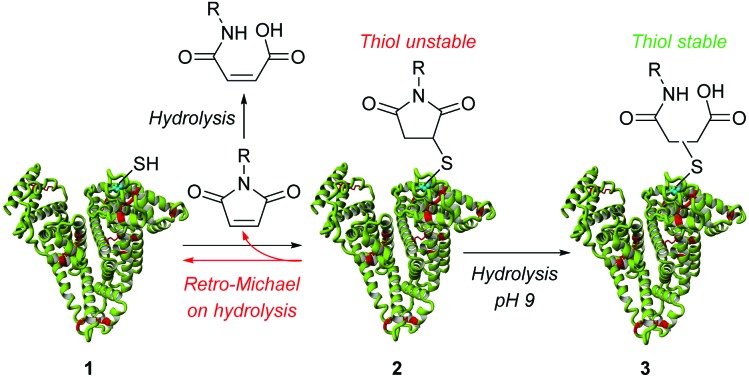
Classical approach to albumin conjugation, highlighting limitations and alternative pathways.

To avoid retro-Michael reactivity, the succinimide may be hydrolysed to succinic acid **3**, effectively locking the conjugate to be thiol-stable.^15^ The property of thiol-stability by hydrolysis is desirable as it would ensure that there was no undesirable thiol transfer taking place in various environments *in vivo*. To this end, we constructed bioconjugate **2** (R = Me) and attempted to selectively hydrolyse the succinimide ring under a range of hydrolysis conditions (*e.g.* temperature and pH). However, the yield of the hydrolysed thiol-stable conjugate was only in the order of 50–60% due to a competing retro-Michael pathway during hydrolysis (see ESI† and Fig. 2). Although retro-Michael deconjugation affords the starting materials initially, the free maleimide is also hydrolysed irreversibly under the reaction conditions, which limits yield (see Scheme 1). Strategies have been developed to address this issue but they require highly specific linkers and their success tends to be protein and protein local microenvironment specific.^15^

**Fig. 2:**
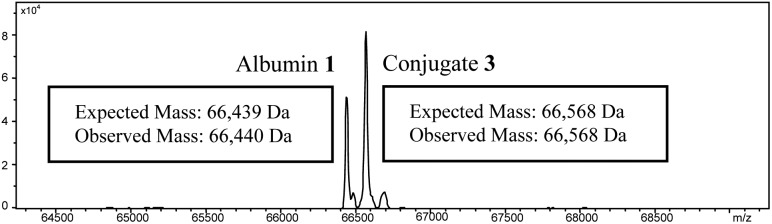
Deconvoluted mass spectrum obtained upon attempted hydrolysis of conjugate **2** at pH 9 showing a mixture of albumin **1** and conjugate **3**.

To supersede conventional maleimide-bioconjugation we would require a moiety that did not suffer from competing retro-Michael mediated deconjugation during hydrolysis whilst retaining the favourable characteristics of efficient and chemoselective reaction with maleimides. To this end, we set about exploring monobromomaleimides in this context.^16^ Our choice of using monobromomaleimides was motivated mainly by the fact that reaction with a thiol proceeds *via* an addition-elimination sequence, *i.e.* rather than addition only. This affords a thioether maleimide motif, for which the retro-Michael pathway is no longer mechanistically feasible (Fig. 3). Our study began by appraising the efficiency and selectivity of conjugation with a monobromomaleimide. We were delighted to find that *N*-methyl monobromomaleimide reacted within the same time-frame and with the same specificity as a classical maleimide (Fig. 3b, see ESI† for further details).

**Fig. 3:**
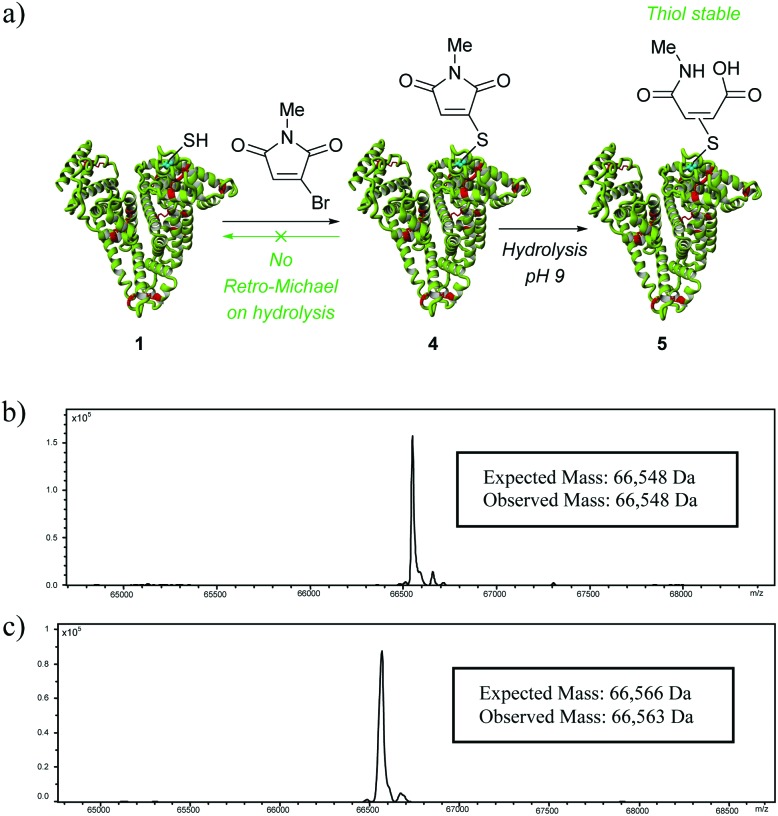
(a) Conjugation of *N*-methyl monobromomaleimide to albumin to form bioconjugate **4**, and subsequent hydrolysis to afford **5**; (b) deconvoluted MS data for maleimide–albumin bioconjugate **4**; (c) deconvoluted MS data for maleamic acid-albumin bioconjugate **5**.

We next treated thioether maleimide conjugate **4** under basic conditions to see if we only observed hydrolysed product **5**. Gratifyingly, this was the only product that was observed under the reaction conditions, thus providing an elegant and simple solution to making a thiol-stable construct on albumin. The rate of hydrolysis was similar to that observed for the succinimide analogue.

To confirm the absence of thiol reactivity of maleamic acid-albumin bioconjugate **5**, it was incubated with 50 equivalents of glutathione (1 mM) at pH 7.4 in PBS (Fig. 4) for 4 h. Consistent with our previous studies, no thiol exchange was observed after incubation.^16*a*^ In fact, no significant transfer was observed even after 95 h. This is in sharp contrast to succinimide bioconjugate **2** (where R = Me), where significant thiol exchange was observed after 4 h (see ESI†, Fig. S15†). Actually, the only succinimide conjugate that remained attached to albumin after this time was the hydrolysed construct, which is known to be thiol stable.^15^

**Fig. 4:**
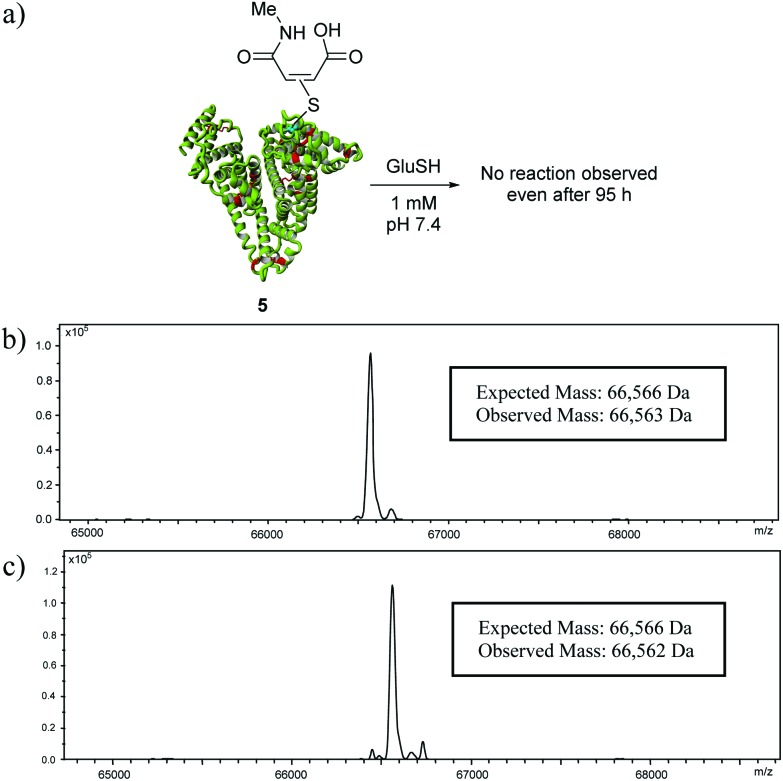
(a) Thiol stability of maleamic acid–albumin bioconjugate **5**; (b) deconvoluted MS data for maleamic acid-albumin bioconjugate **5** after 4 hours of incubation; and (c) deconvoluted MS data for maleamic acid-albumin bioconjugate **5** after 95 hours of incubation.

Following our work on developing a thiol stable construct, we set about incorporating simple, modular ‘click’ chemistry into our strategy through the use of *N*-propargyl monobromomaleimide and Alexa Fluor® 488 azide (see Scheme 2, see ESI† for further details). If successful, this would result in a facile method for forming various thiol-stable functional bioconjugates. Pleasingly, clicking *N*-propargyl monobromomaleimide with Alexa Fluor® 488 azide followed by conjugation to albumin **1** afforded bioconjugate **6** by MS and UV-Vis absorption. This species was then hydrolysed to thiol-stable bioconjugate **7** without any deconjugation, thus highlighting how our platform can incorporate a ‘click’ modification strategy.

**Scheme 2:**
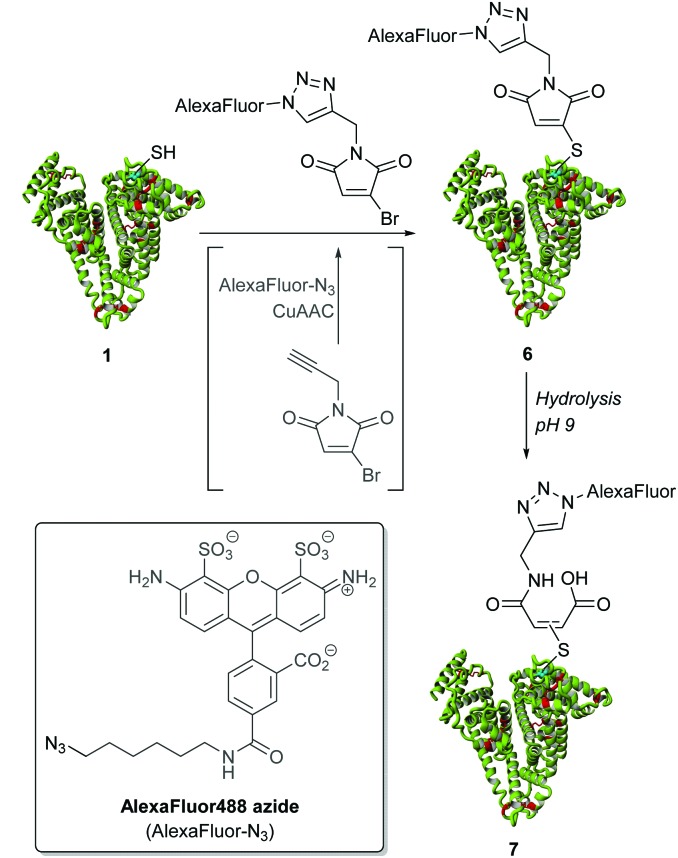
Click functionalisation strategy for creating thiol-stable albumin conjugate **7**.

## Conclusions

In conclusion, an elegant, robust, high yielding and thiol-stable alternative to classical maleimide conjugation to human serum albumin has been described. Classical maleimide conjugation has been shown to be reversible and methods for hydrolysis to thiol-stable thioether succinimides were shown to be unsuccessful as they led to significant retro-Michael mediated deconjugation. The use of monobromomaleimides results in rapid and selective conjugation, and hydrolysis leads to thiol-stable maleamic acid only, due to the absence of a retro-Michael pathway mechanistically. The exemplification of the chemistry *via* a ‘click’ strategy highlights how it may be readily utilised in various applications in a rapid manner. As well as providing a general, efficient approach to creating thiol-stable cysteine conjugates, this works sets the foundation for a platform for half-life extension by the use of stable human serum albumin conjugation.
