# Supplementary material for: A platform for efficient, thiol-stable conjugation to albumin's native single accessible cysteine
Source: Org Biomol Chem. 2015 Jun 25;13(29):7831–2. doi: 10.1039/c5ob01205h (PMC4563668; doi:10.1039/c5ob01205h)
Supplement: Supplementary file 1 [file OB-013-C5OB01205H-s001.pdf]

Electronic Supplementary Material (ESI) for Organic & Biomolecular Chemistry.
This journal is © The Royal Society of Chemistry 2015

A platform for efficient, thiol-stable conjugation to albumin's native single accessible cysteine

Mark E. B. Smith,[a] Mikael B. Caspersen,[b] Eifion Robinson,[a] Maurício Morais,[a] Antoine Maruani,[a] João P. M. Nunes,[a] Karl Nicholls,[b] Malcolm J. Saxton,[b] Stephen Caddick,[a] James R. Baker*[a] and Vijay Chudasama*[a]

[a] *Department of Chemistry, University College London, London, WC1H 0AJ, UK.* [b] *Novozymes Biopharma UK Ltd, Castle Court, 59 Castle Boulevard, Nottingham, UK, NG7 1FD.*

** Tel.: +44 (0)20 7679 2653 and +44 (0)20 7679 2077; E-mail: j.r.baker@ucl.ac.uk and v.chudasama@ucl.ac.uk*

General Experimental

All reagents were purchased from Sigma-Aldrich or Life Technologies and were used as received. Where described below pet. refers to petroleum ether (40–60 °C). All reactions were monitored by thin-layer chromatography (TLC) on pre-coated SIL G/UV_{254} silica gel plates (254 μm) purchased from VWR. Flash column chromatography was carried out with Kiesegel 60 M, 0.04/0.063 mm (200–400 mesh) silica gel. ^{1}H and ^{13}C NMR spectra were recorded at ambient temperature on a Bruker Avance 600 instrument operating at a frequency of 600 MHz for ^{1}H and 150 MHz for ^{13}C in $CDCl_3$. The chemical shifts (δ) for ^{1}H and ^{13}C are quoted relative to residual signals of the solvent on the ppm scale. ^{1}H NMR peaks are reported as singlet (s), doublet (d), triplet (t), quartet (q), quintet (quint.), sextet (sext.), octet (oct.), multiplet (m), broad (br), doublet of doublets (dd), doublet of triplets (dt), AB quartet (ABq). Coupling constants (*J* values) are reported in Hertz (Hz) and are H-H coupling constants unless otherwise stated. Signal multiplicities in ^{13}C NMR were determined using the distortionless enhancement by phase transfer (DEPT) spectral editing technique. Infrared spectra were obtained on a Perkin Elmer Spectrum 100 FTIR Spectrometer operating in ATR mode with frequencies given in reciprocal centimetres (cm^{-1}). Melting points were measured with a Gallenkamp apparatus and are uncorrected. Mass spectra were obtained on a VG70-SE mass spectrometer. Recombinant native sequence albumin **1** was obtained from Novozymes Biopharma.

Protein GPC

Gel permeation chromatography was performed on protein samples using a TSK G3000 SW_{XL} analytical column, 7.8 mm id x 3000 mm length (Tosoh Bioscience) with a TSK SW_{XL} guard column, 6.0 mm id x 40 mm length (Tosoh Bioscience) kept at 30 °C. The analysis was performed on a LC system with LC-10AV pumps, SIL-HT autoinjector, SPD-10AV UV-Vis detector, DGU-14A and a CTO-10AC column oven (Shimadzu). Elution was done with 25 mM sodium phosphate, 0.1 M sodium sulfate, 0.05% sodium azide at 1 mL/min flow rate. Detection was done at 280 nm and calibration for protein concentration determination was performed with 25 µL of an albumin standard of known concentration. The system and analysis was controlled using the Class VP Chromatography software (Shimadzu).

Protein LC-MS (for Figures S1-S17)

Liquid chromatography was performed on protein samples using a Waters Acquity (Waters) with a BEH 50 × 2.1 mm ACQUITY BEH 1.7 µm C4 column (Waters) employing a 5 min 0-70% Acetonitrile

(Rathburn) / water analytical gradient with 0.1% formic acid at a flow rate of 0.4 mL/min. Eluted proteins were directly introduced to a Bruker MicrOTOF II mass spectrometer (Bruker Daltonics) *via* an ESI source. MS mode: ES+. Scan range (*m/z*): 625–3000. Data obtained in continuum mode. The electrospray source of the MS was operated with a capillary voltage of 4.5 kV and a capillary exit voltage of 160 V. Nitrogen was used as the nebulizer and dry gas at a 3.0 bar pressure for the nebulizer gas and 7.0 L/min for the dry gas. Ion series were generated by integration of the total ion chromatogram (TIC) over the 1.5–2.0 min range. Total mass spectra for protein samples were reconstructed from the ion series using the Compass DataAnalysis software using a baseline subtraction of 0.4 and a Gaussian smoothing with a width of 0.076 Da before deconvolution. All instrument control and sample tables were controlled using BioPharma Compass™ (Bruker Daltonics).

Protein LC-MS (for Figures S18-S19)

LC-MS was performed on protein samples using a Thermo Scientific uPLC connected to MSQ Plus Single Quad Detector (SQD). Column: Hypersil Gold C4, 1.9 µm, 2.1 × 50 mm. Wavelength: 254 nm. Mobile Phase: 99:1 Water (0.1% formic acid): MeCN (0.1% formic acid) to 1:9 Water (0.1% formic acid): MeCN (0.1% formic acid) gradient over 4.5 min. Flow Rate: 0.3 mL/min. MS Mode: ES+. Scan Range: m/z = 500–2000. Scan time: 1.5 s. Data obtained in continuum mode. The electrospray source of the MS was operated with a capillary voltage of 3.5 kV and a cone voltage of 50 V. Nitrogen was used as the nebulizer and desolvation gas at a total flow of 600 L/h. Ion series were generated by integration of the total ion chromatogram (TIC) over the 3.3-3.8 min range. Total mass spectra for protein samples were reconstructed from the ion series using the pre-installed ProMass software using default settings for large proteins in m/z range 500–1500.

Cloning and expression of proteins

Albumin 1

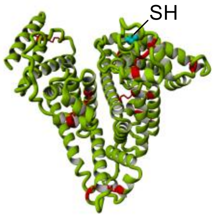

1

Native sequence albumin, AlbIX, with >90% free thiol for cysteine-34. Expected mass: 66,439 Da (albumin **1** with cysteine in reduced form). Observed mass: 66,441 Da.

(a)

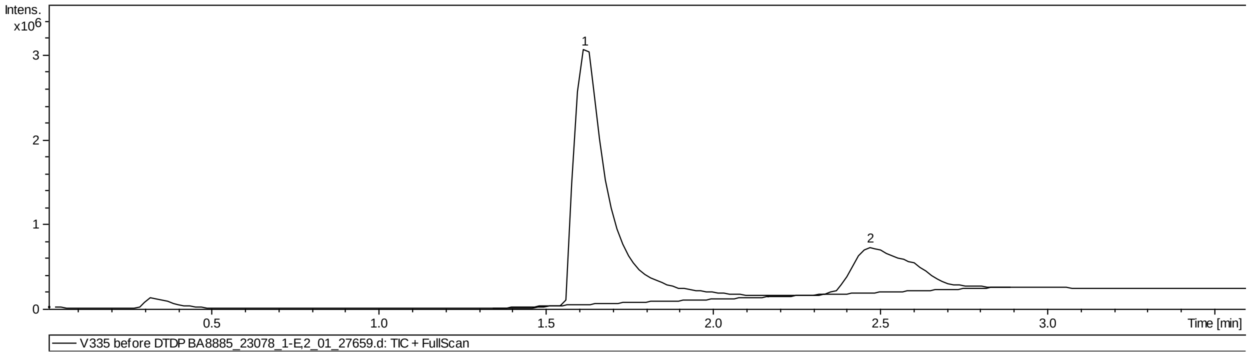

(b)

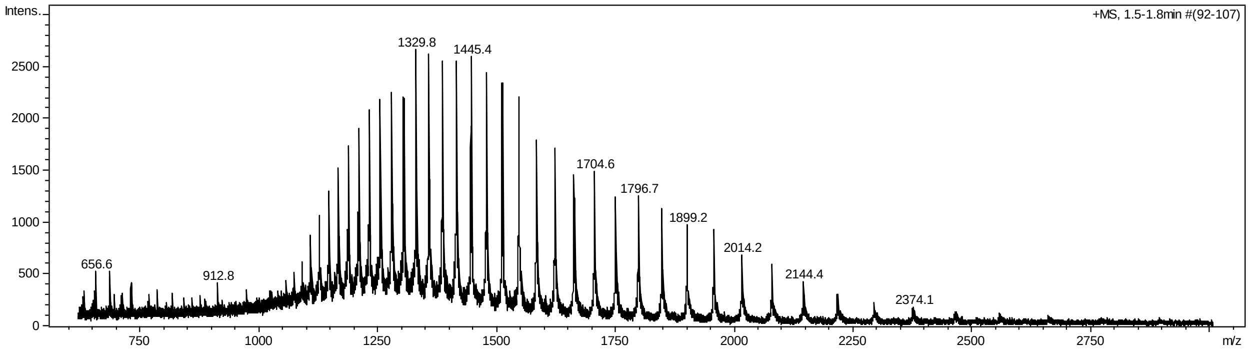

(c)

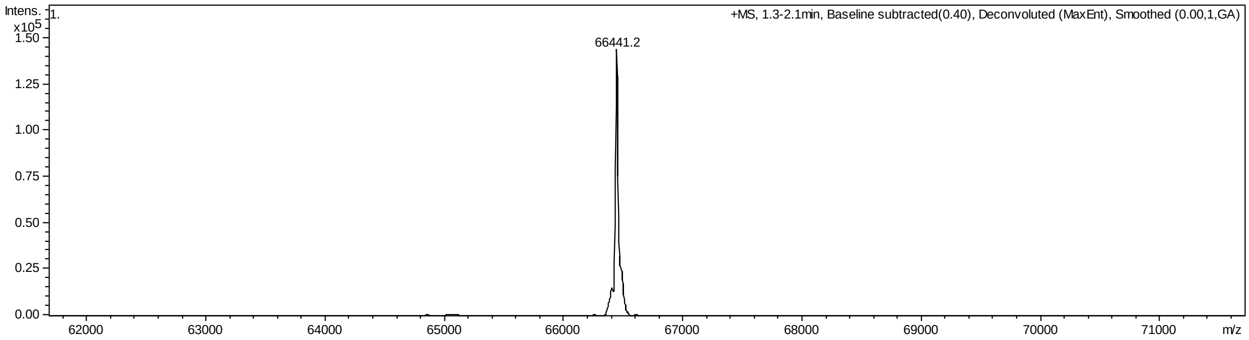

Figure S1. (a) TIC, (b) non-deconvoluted and (c) deconvoluted MS data for albumin **1**.

Synthesis of compounds

3-Bromo-1-methyl-1*H*-pyrrole-2,5-dione[1]

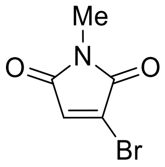

To a solution of *N*-methylmaleimide (2.87 g, 25.0 mmol) in $CHCl_3$ (125 mL) was added bromine (2.60 mL, 55.5 mmol) dropwise and the resulting mixture was refluxed for 2 h. After this time, the solvent was removed *in vacuo* and the resulting solid dissolved in EtOAc (30 mL) and washed with 15% aq. $Na_2S_2O_3$ (20 mL). The product was extracted with EtOAc (3 x 30 mL), dried ($MgSO_4$) and the solvent was removed *in vacuo* to afford crude 2,3-dibromosuccinimide as yellow crystals. The succinimide product was dissolved in AcOH (150 mL), to which was added sodium acetate (5.94 g, 71.6 mmol) and the reaction mixture was refluxed for 2.5 h. After this time, the reaction mixture was left to cool down to room temperature before the solvent was removed *in vacuo*. The crude mixture was dissolved in EtOAc (50 mL) and washed with sat. aq. Na_2CO_3 (3 x 25 mL). The organic phase was dried ($MgSO_4$) and the solvent removed *in vacuo*. Purification by flash chromatography (Pet/EtOAc (20-40%)) afforded 3-bromo-1-methyl-1*H*-pyrrole-2,5-dione (2.86 g, 15.1 mmol, 65%) as white crystals: mp 100-101 °C (lit. mp 88-89 °C[1]); IR (solid) 3105, 2947, 1776, 1704, 1588, 1493 cm^{-1}; ^{1}H NMR (600 MHz, $CDCl_3$) δ 6.88 (1H, s), 3.07 (3H, s); ^{13}C NMR (150 MHz, $CDCl_3$) δ 168.7 (C), 165.5 (C), 132.0 (CH), 131.5 (C), 24.8 (CH_3); *m/z* (CI$^+$) 192 (100%, [^{81}M+H]$^+$), 190 (100%, [^{79}M+H]$^+$); HRMS (CI$^+$) $C_5H_5{}^{79}BrNO_2$ ([M+H]$^+$) calcd. 189.9504, found 189.9501.

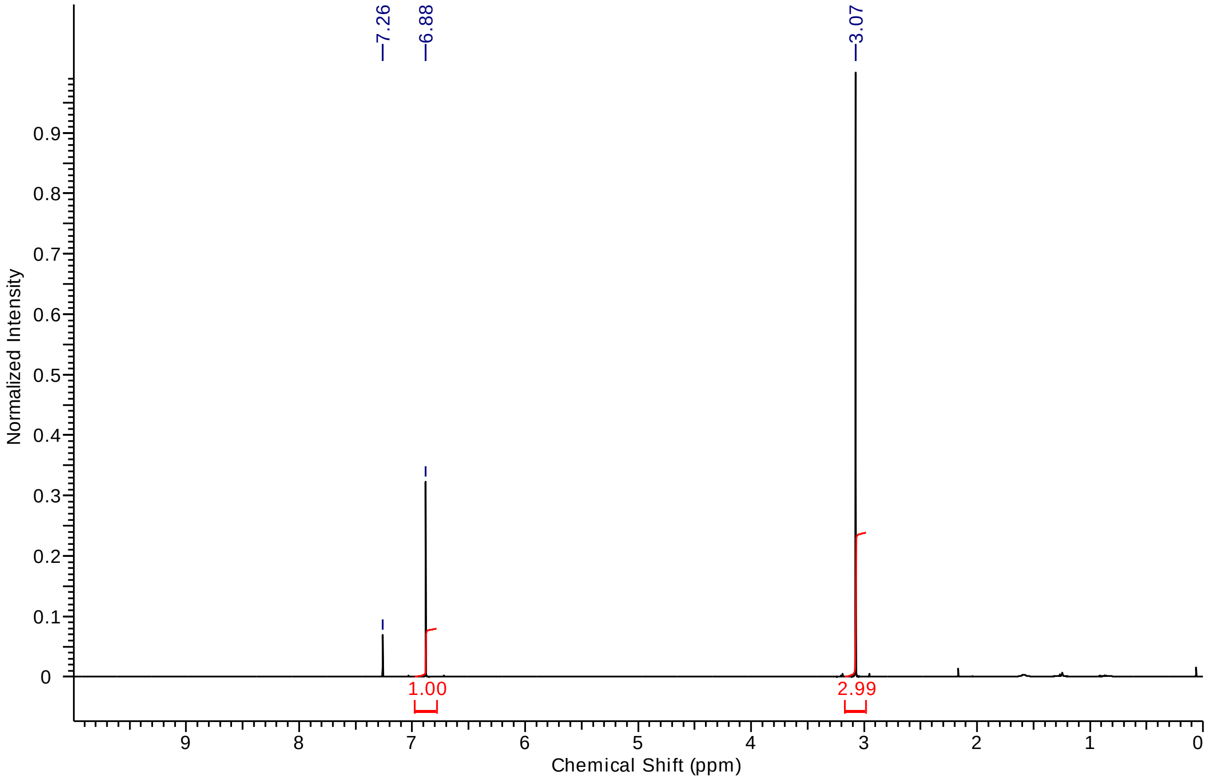

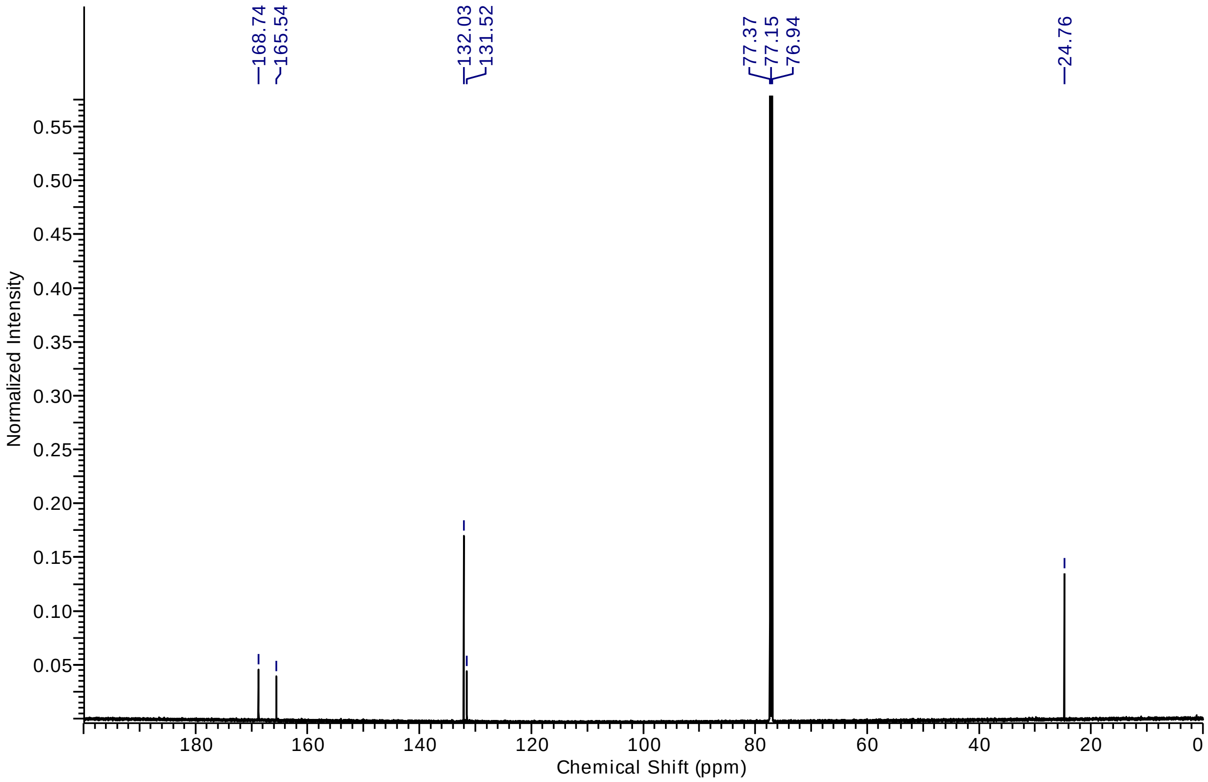

3-Bromo-1-(prop-2-yn-1-yl)-1*H*-pyrrole-2,5-dione

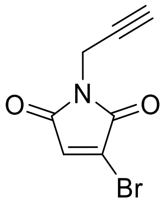

To a solution of bromomaleic anhydride (0.40 g, 2.3 mmol) in AcOH (5 mL) was added propargylamine (144 µL, 2.5 mmol) and the resulting mixture heated under refluxed for 6 h. The solvent removed *in vacuo* and traces of AcOH were co-evaporated with toluene. The resulting solid was dry loaded on silica and purified by flash chromatography (Pet/EtOAc (20-40%)) to afford 3-bromo-1-(prop-2-yn-1-yl)-1*H*-pyrrole-2,5-dione (0.40 g, 1.80 mmol, 83%) as white crystals: mp 104-105 °C IR (solid) 3272, 3095, 2132, 1771, 1702, 1587 cm^{-1}; ^{1}H NMR (600 MHz, $CDCl_3$) δ 6.93 (1H, s), 4.33 (2H, d, *J* = 3.6 Hz), 2.24 (1H, t, *J* = 3.6 Hz); ^{13}C NMR (150 MHz, $CDCl_3$) δ 167.1 (C), 164.0 (C), 132.2 (CH), 131.8 (C), 76.8 (C), 72.1 (CH), 27.8 (CH_2). *m/z* (CI^+) 216 (100%, $[^{81}M+H]^+$), 214 (100%, $[^{79}M+H]^+$); HRMS (CI+) $C_7H_5{}^{79}BrNO_2$ ($[M+H]^+$) calcd. 213.9425, found 213.9429.

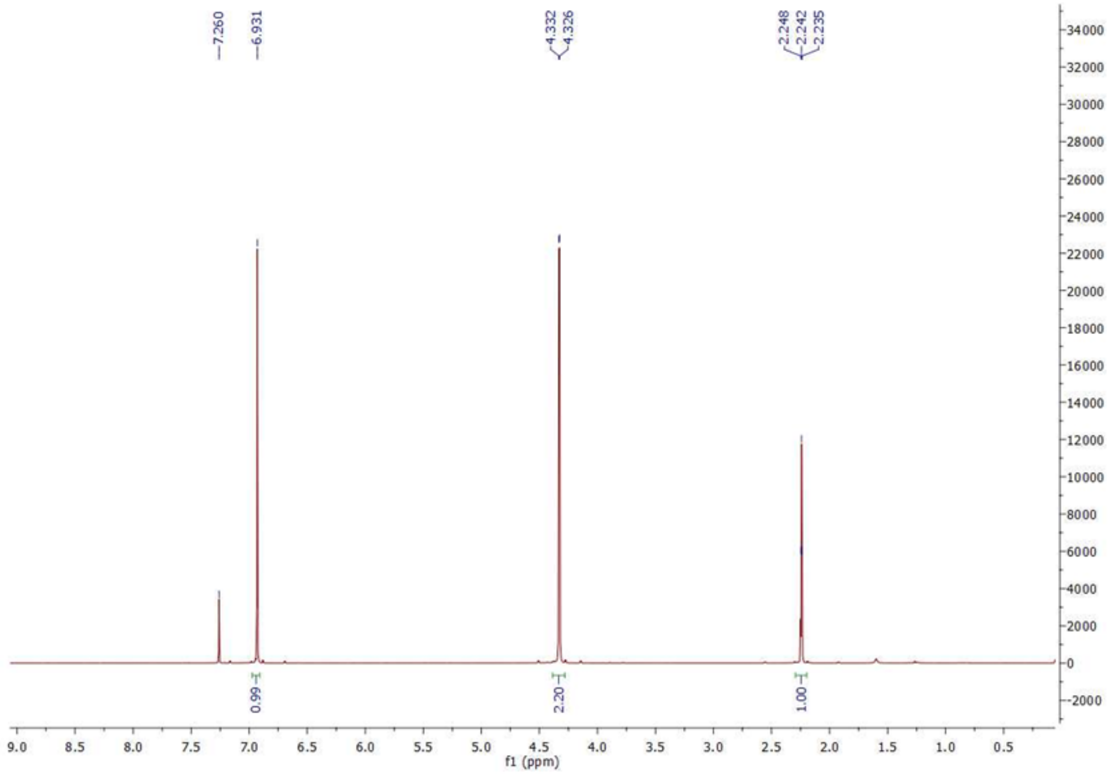
7.260
6.931
4.332
4.326
2.248
2.242
2.235
0.99
2.20
1.00
f1 (ppm)

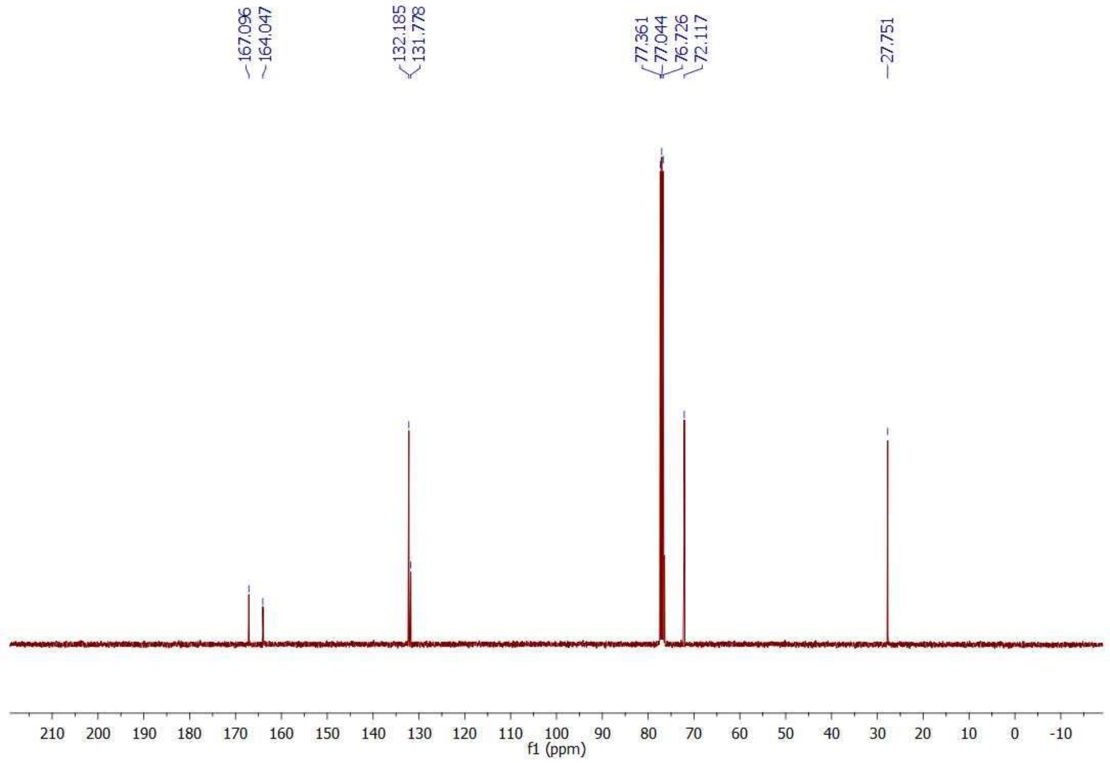
167.096
164.047
132.185
131.778
77.361
77.044
76.726
72.117
27.751
f1 (ppm)

Bioconjugation reactions involving human serum albumin

Reaction of human serum albumin with *N*-methyl maleimide

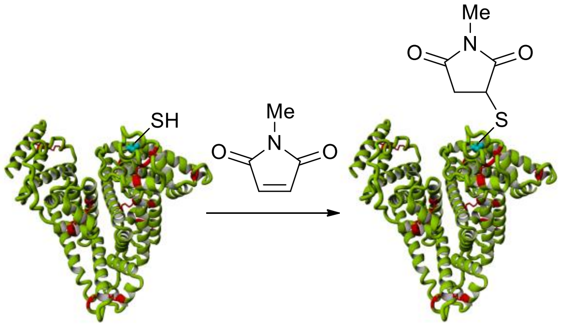

1 **2**

As >90% of cysteine-34 of albumin is in its reduced form, no pre-treatment was necessary. To albumin **1** (900 µL, 209 µM, 188 nmol) in phosphate buffered saline (PBS) (pH 7.4) was added a freshly prepared solution of *N*-methyl maleimide (100 µL, 2.82 mM, 282 nmol) in PBS (pH 7.4), and the reaction mixture incubated at room temperature in the dark for 3 hours. The reaction was desalted using a PD MidiTrap G-25 (GE Healthcare) following the manufacturer's instructions. The sample was analysed by GPC for concentration (data not shown) and LC-MS. Expected mass: 66,550 Da (bioconjugate **2**). Observed mass: 66,552 Da.

(a)

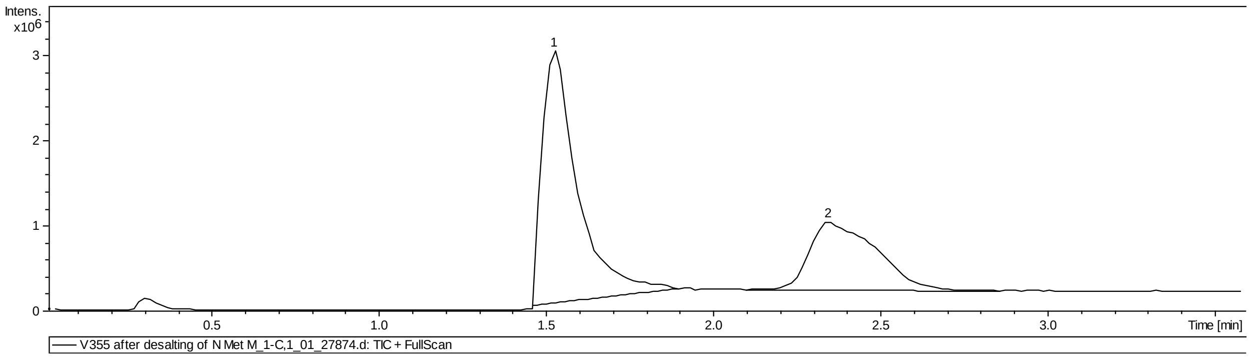

(b)

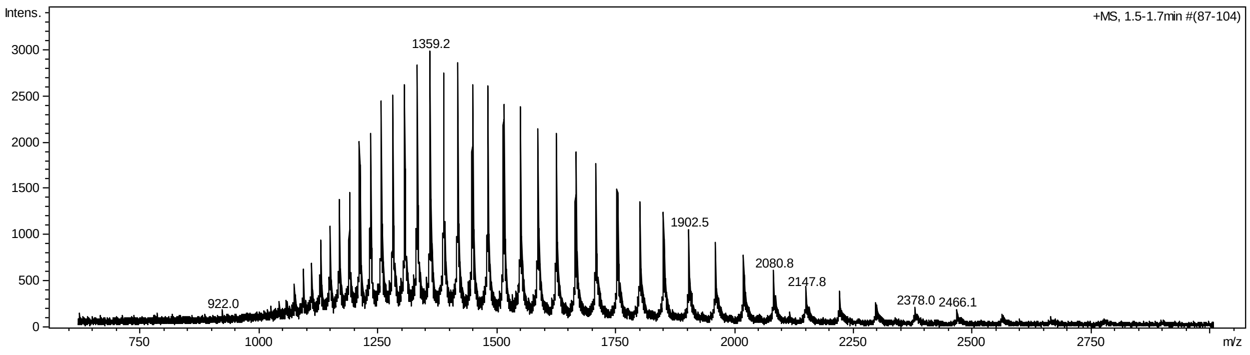

(c)

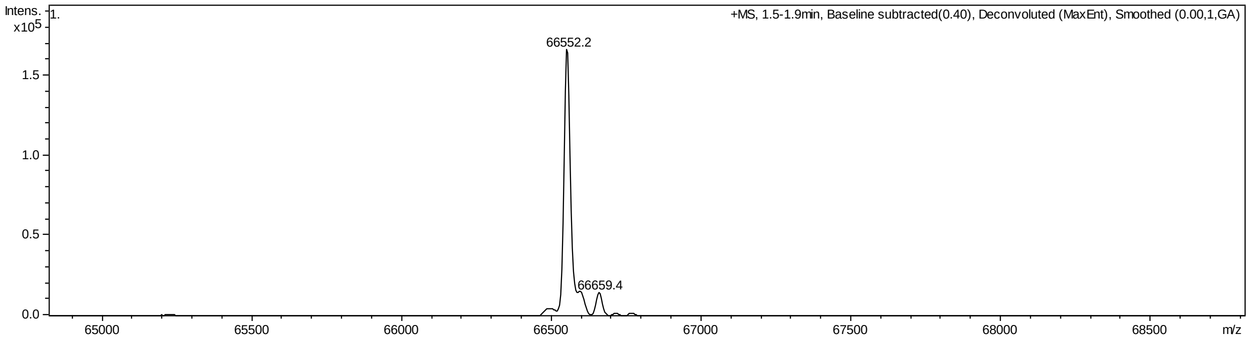

Figure S2 (a) TIC, (b) non-deconvoluted and (c) deconvoluted MS data for formation of bioconjugate **2**.

Attempted hydrolysis of succinimide bioconjugate 2

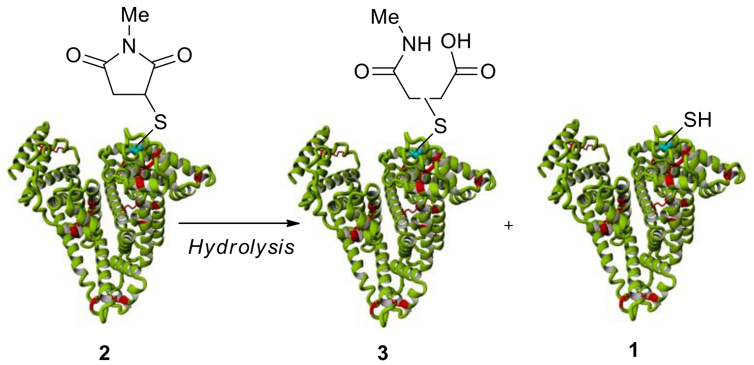

Hydrolysis was carried out by adding glycine (250 mM stock) to *N*-methyl succinimide albumin conjugate **2** (25 mM final concentration), adjusting the pH to pH 9 with 0.5 M NaOH and incubating overnight at 37 °C. The resulting conjugate was placed in fresh PBS (pH 7.4) using a PD MidiTrap G-25 (GE Healthcare) following the manufacturer's instructions. The sample was analysed by GPC for concentration and LC-MS. Expected mass: 66,568 Da (bioconjugate **3**), 66,439 Da (albumin **1**). Observed masses: 66,440 Da at approximately 40% signal intensity relative to observed mass at 66,568 at approximately 60% signal intensity.

(a)

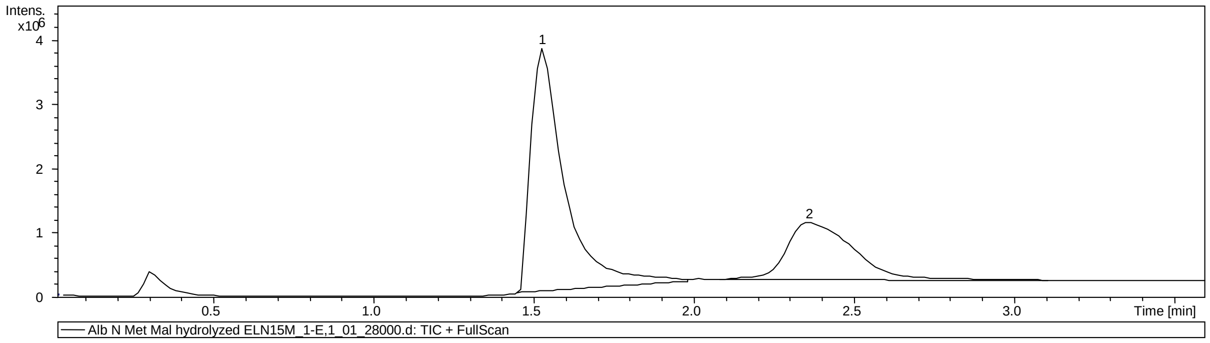

(b)

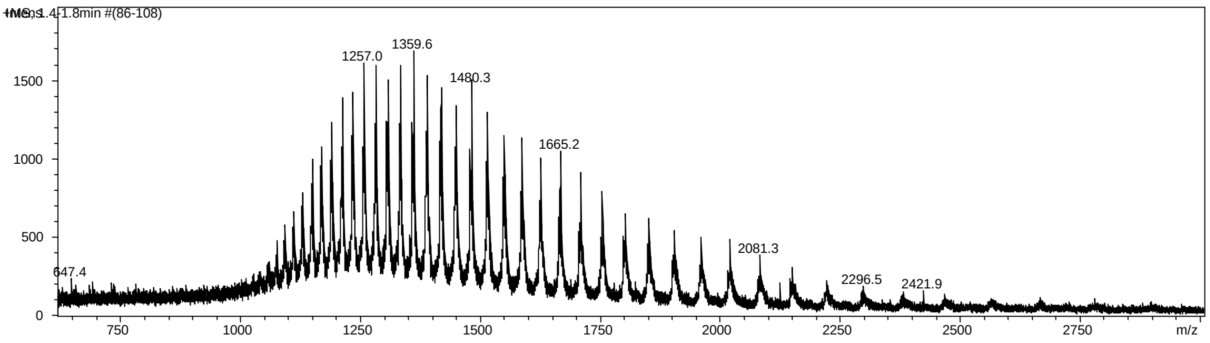

(c)

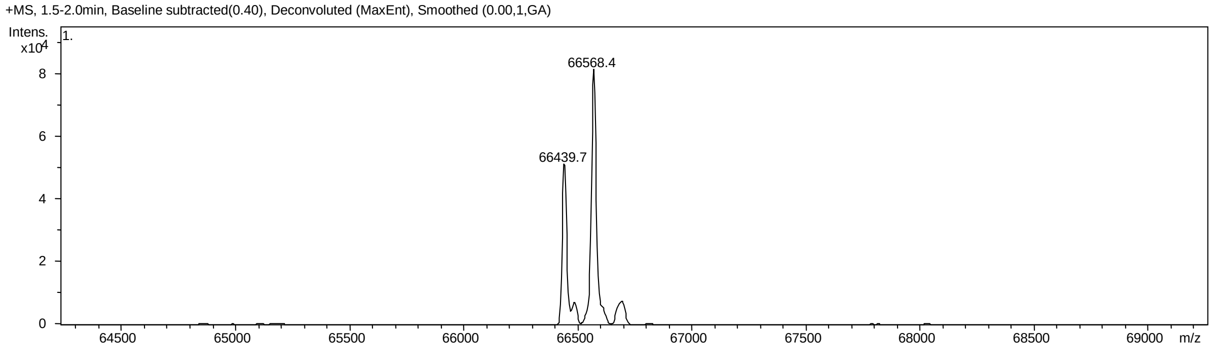

Figure S3. (a) TIC, (b) non-deconvoluted and (c) deconvoluted MS data for attempted hydrolysis of bioconjugate **2**.

Reaction of human serum albumin with 3-bromo-1-methyl-1*H*-pyrrole-2,5-dione

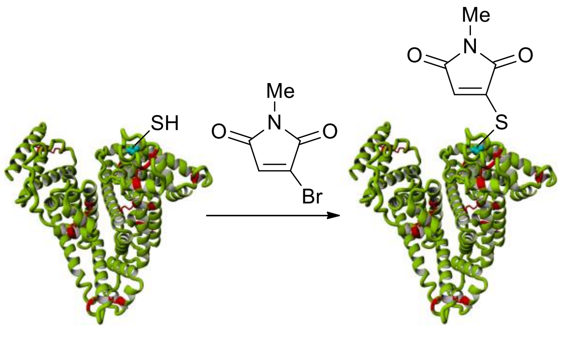

1 **4**

As >90% of cysteine-34 of albumin is in its reduced form, no pre-treatment was necessary. To albumin **1** (900 µL, 209 µM, 188 nmol) in PBS (pH 7.4) was added a freshly prepared solution of 3-bromo-1-methyl-1*H*-pyrrole-2,5-dione (100 µL, 2.82 mM, 282 nmol) in PBS (pH 7.4), and the reaction mixture incubated at room temperature in the dark for 3 hours. The reaction was desalted using a PD MidiTrap G-25 (GE Healthcare) following the manufacturer's instructions. The sample was analysed by GPC for concentration and LC-MS. Expected mass: 66,548 Da (bioconjugate **4**). Observed mass: 66,548 Da.

(a)

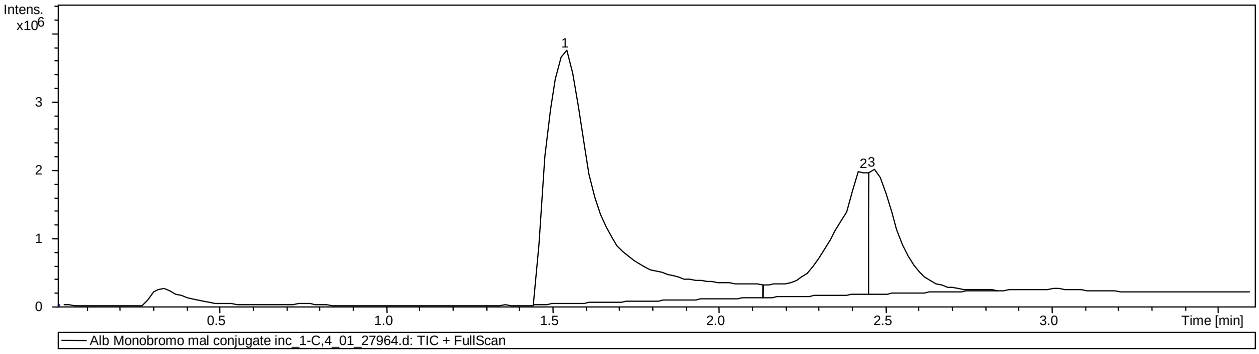

(b)

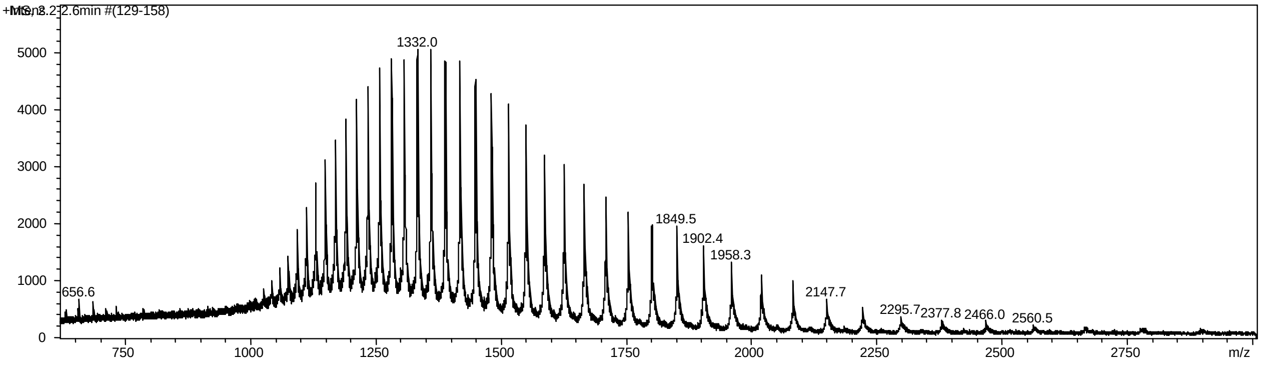

(c)

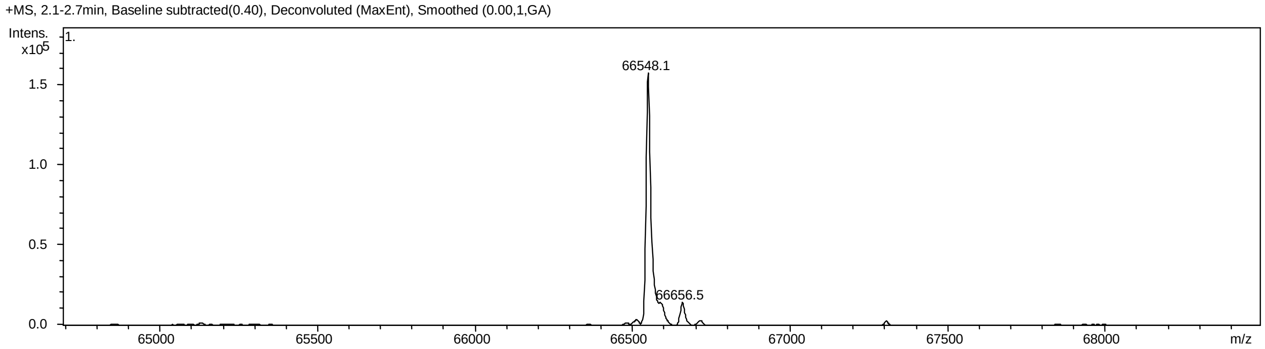

Figure S4. (a) TIC, (b) non-deconvoluted and (c) deconvoluted MS data for formation of bioconjugate **4**.

Attempted hydrolysis of succinimide bioconjugate 4

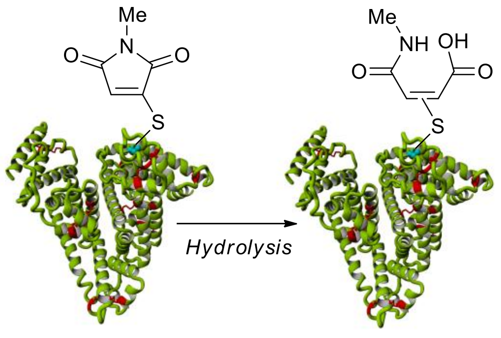

4 **5**

Hydrolysis of *N*-methyl maleimide albumin conjugate **4** was achieved by adding glycine (25 mM final concentration), adjusting the pH to pH 9 with 0.5 M NaOH, and incubating overnight at 37 °C. The bioconjugate **5** was placed in fresh PBS (pH 7.4) using a PD MidiTrap G-25 (GE Healthcare) following the manufacturer's instructions. The sample was analysed by GPC for concentration and LC-MS. Expected mass: 66,439 Da (albumin **1**), 66,566 Da (bioconjugate **5**). Observed mass: 66,563 Da.

(a)

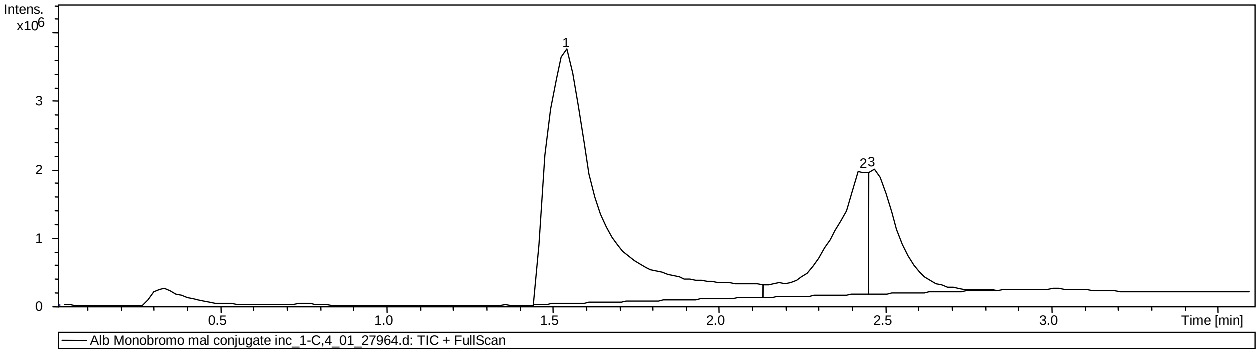

(b)

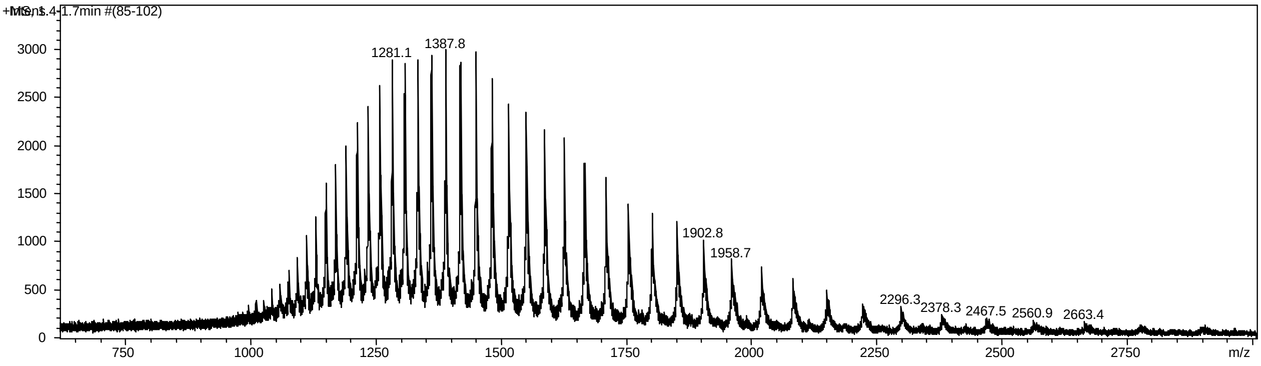

(c)

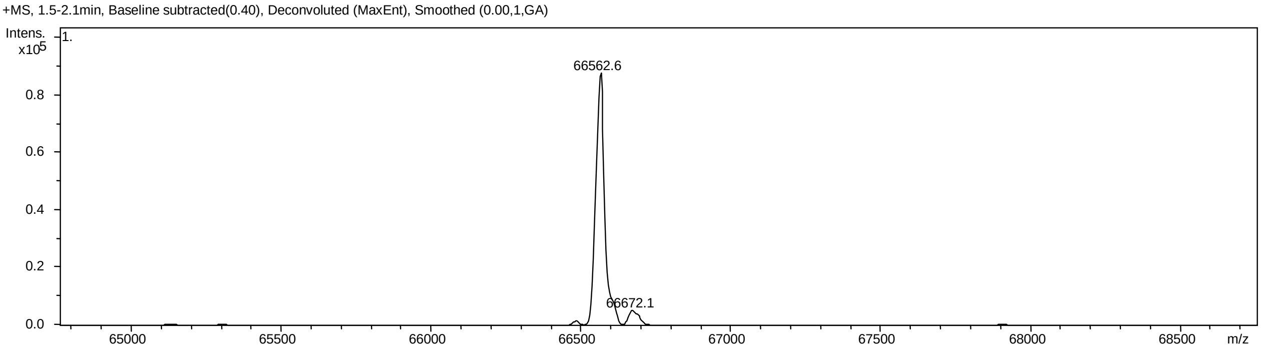

Figure S5. (a) TIC, (b) non-deconvoluted and (c) deconvoluted MS data for attempted hydrolysis of bioconjugate **4**.

Stability experiment with bioconjugate 5 in PBS

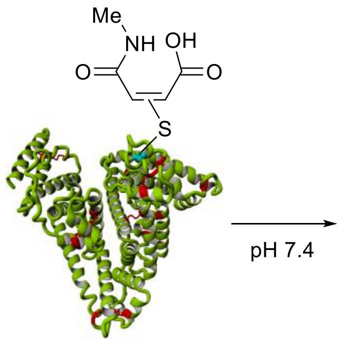

5

Bioconjugate **5** was diluted with PBS (pH 7.4) to give a final concentration of 19.5 μM. This sample was placed at 37 °C protected from light. The sample was analysed by LC-MS at different times. Expected mass: 66,566 Da (bioconjugate **5**). Observed mass: 66,563 Da at 4 hours and 66,562 Da at 42 hours and 66,561 Da 95 hours.

4 hours

(a)

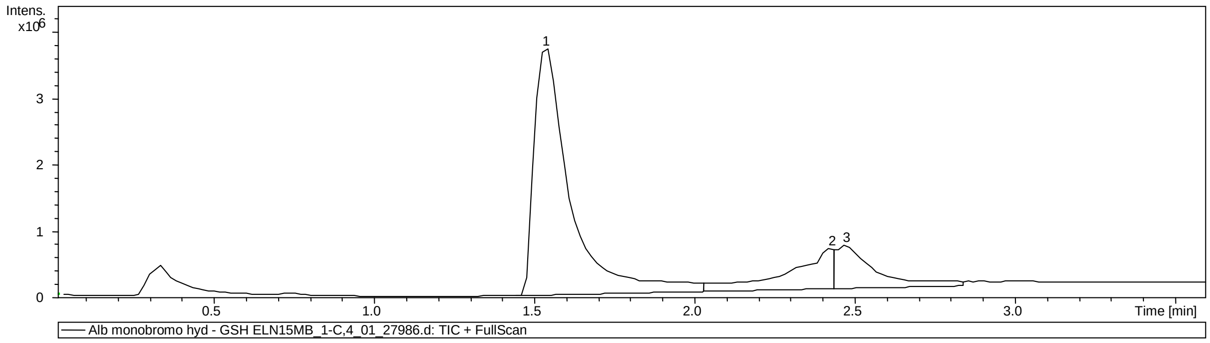

(b)

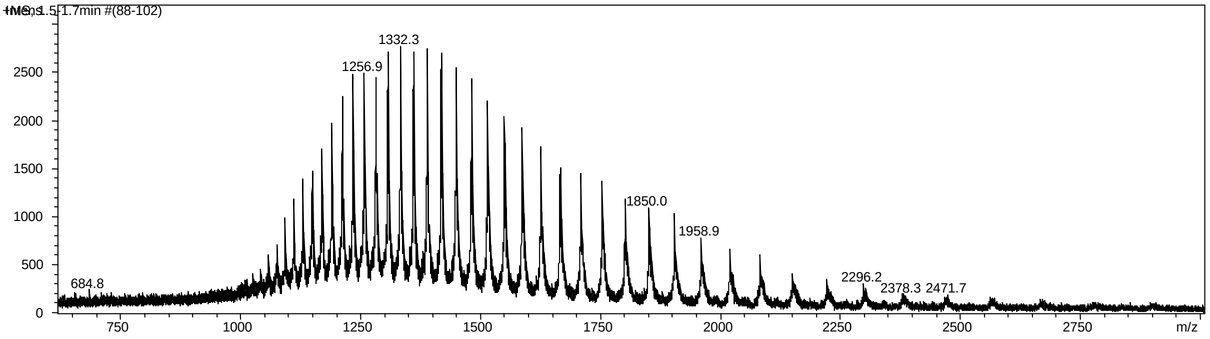

(c)

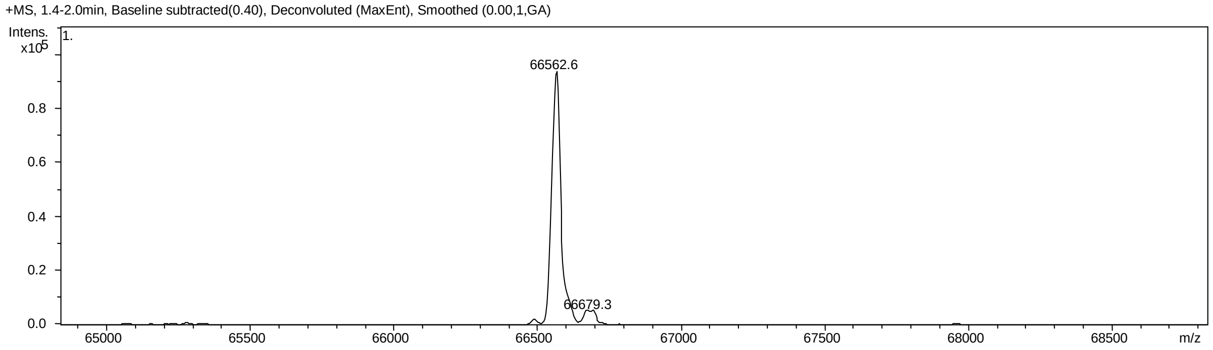

Figure S6. (a) TIC, (b) non-deconvoluted and (c) deconvoluted MS data for stability experiment with bioconjugate **5** after 4 h.

42 hours

(a)

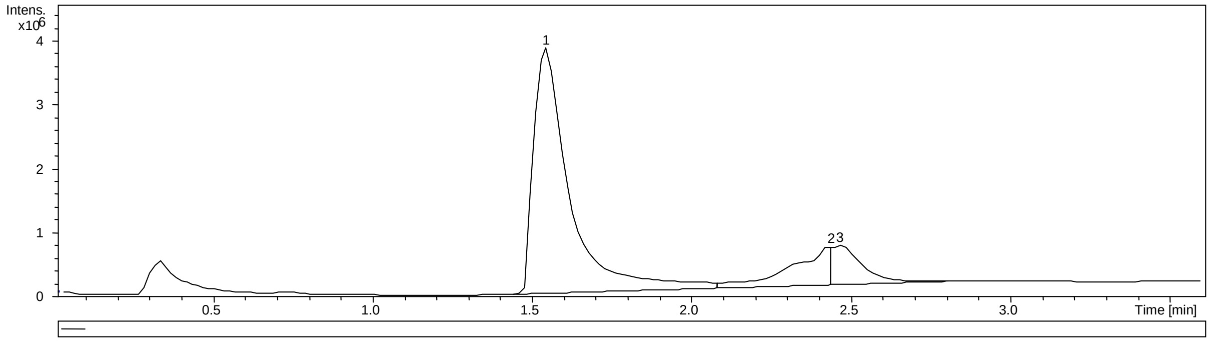

(b)

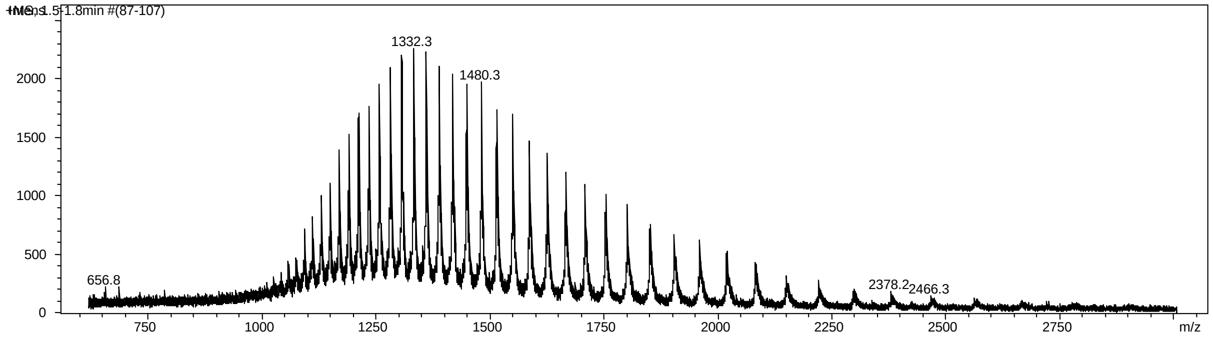

(c)

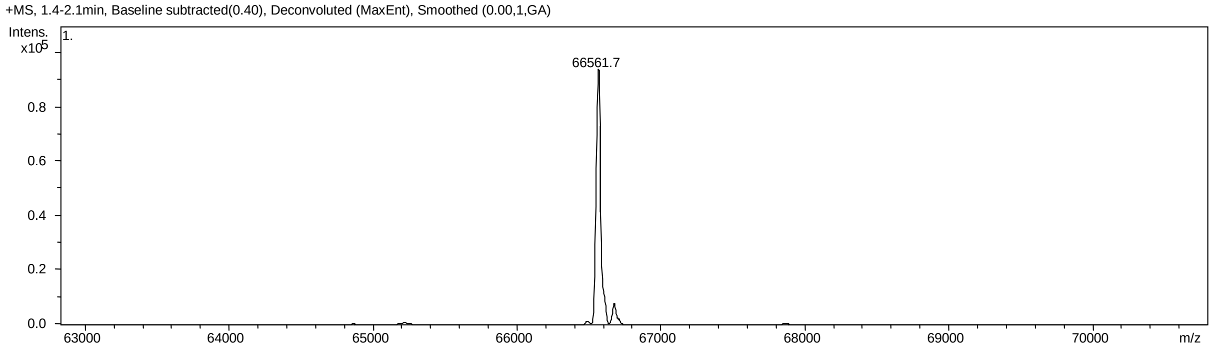

Figure S7. (a) TIC, (b) non-deconvoluted and (c) deconvoluted MS data for stability experiment with bioconjugate **5** after 42 h.

95 hours

(a)

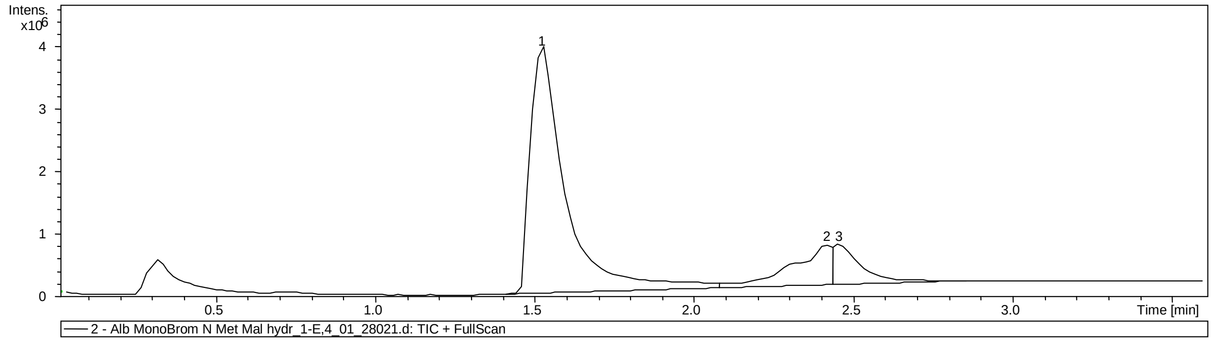

(b)

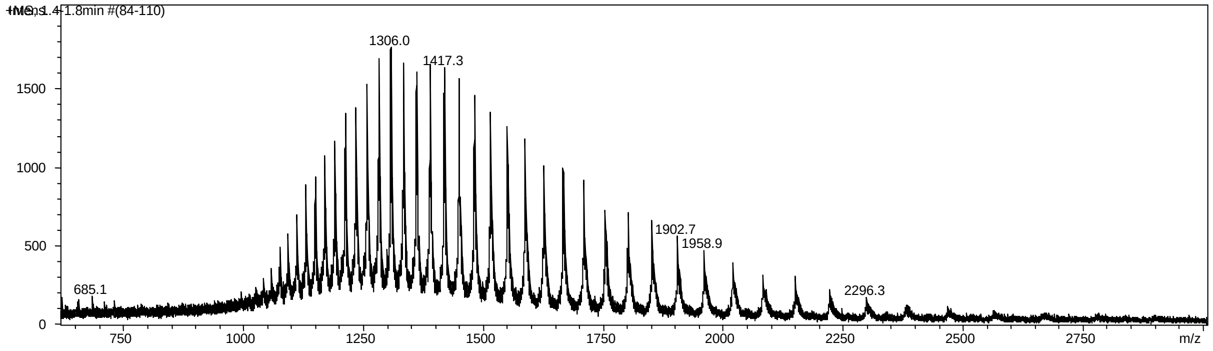

(c)

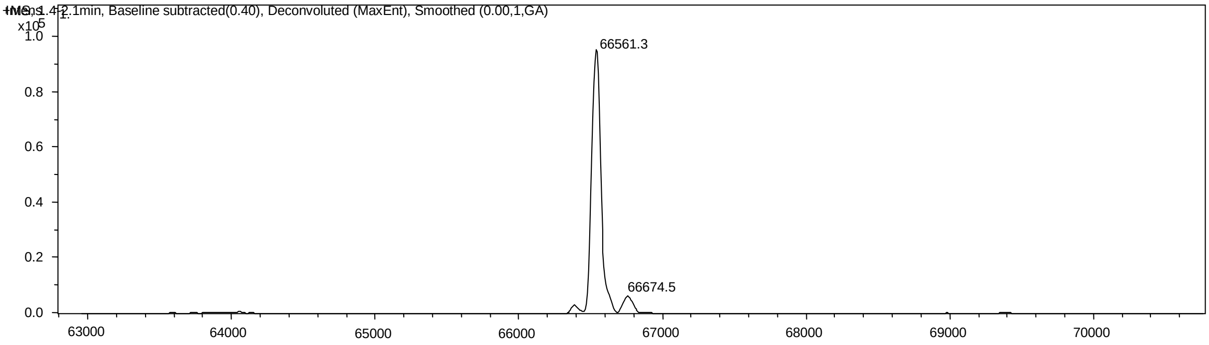

Figure S8. (a) TIC, (b) non-deconvoluted and (c) deconvoluted MS data for stability experiment with bioconjugate **5** after 95 h.

Stability experiment with bioconjugate 5 in PBS with Thiol

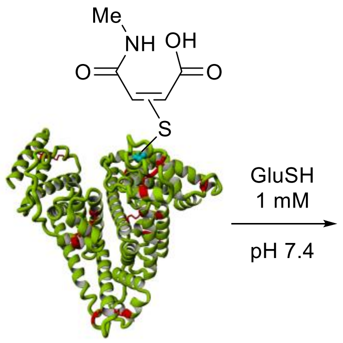

5

Bioconjugate **5** (53 µL, 37.6 µM, 2 nmol) was diluted to a final concentration of 20 µM by adding PBS (pH 7.4) (37 µL) and a solution of GluSH (10 µL, 10 mM, 100 nmol) in PBS (pH 7.4) to give a 50 times excess of GluSH to albumin. This sample was placed at 37 °C protected from light. The sample was analysed by LC-MS at different times. Expected mass: 66,439 Da (albumin **1**), 66,566 Da (bioconjugate **5**). Observed mass: 66,563 Da at 4 hours and 66,562 at both 42 hours and 95 hours.

4 hours

(a)

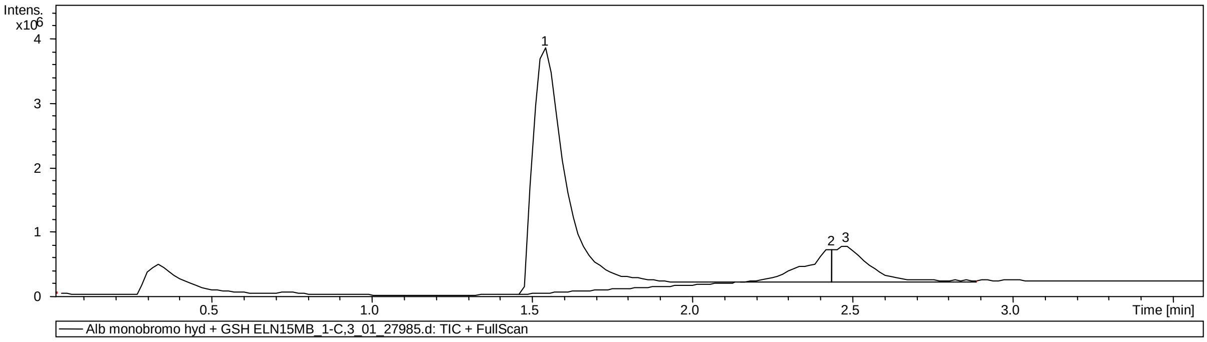

(b)

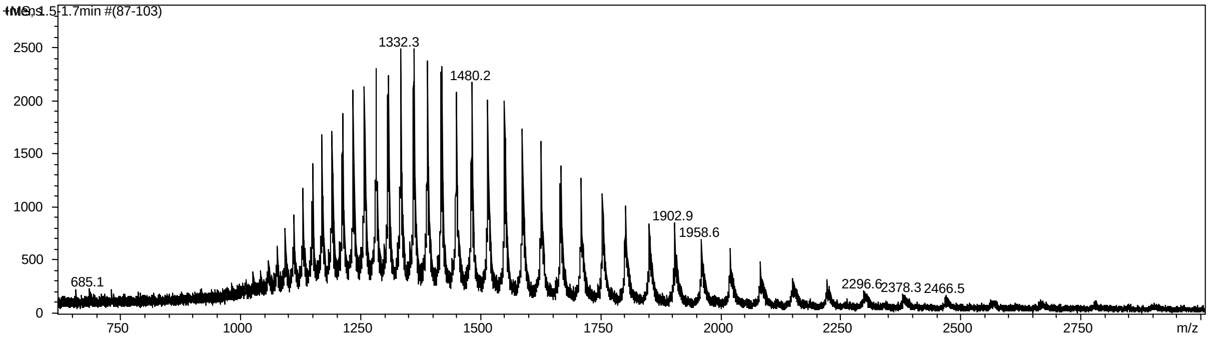

(c)

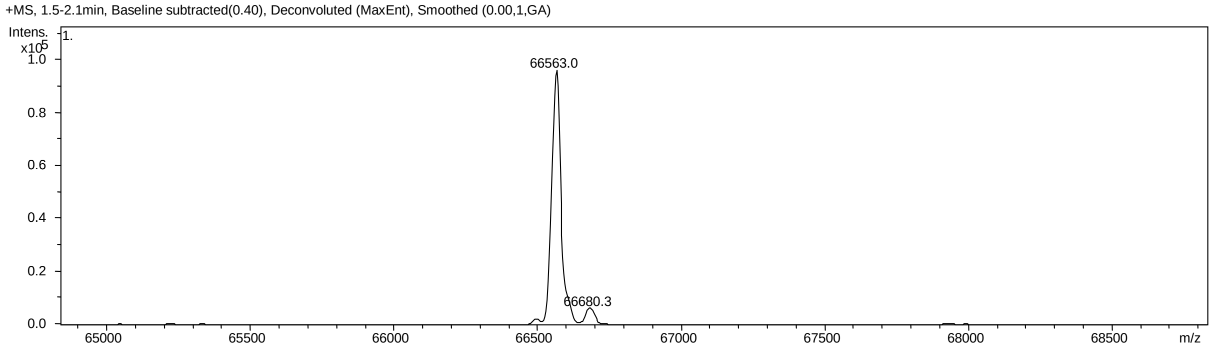

Figure S9. (a) TIC, (b) non-deconvoluted and (c) deconvoluted MS data for attempted thiol exchange from bioconjugate **5** after 4 h.

42 hours

(a)

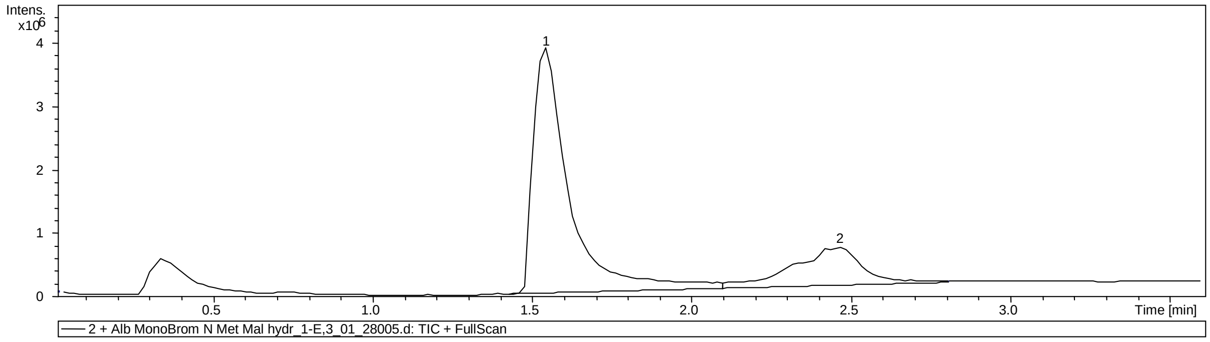

(b)

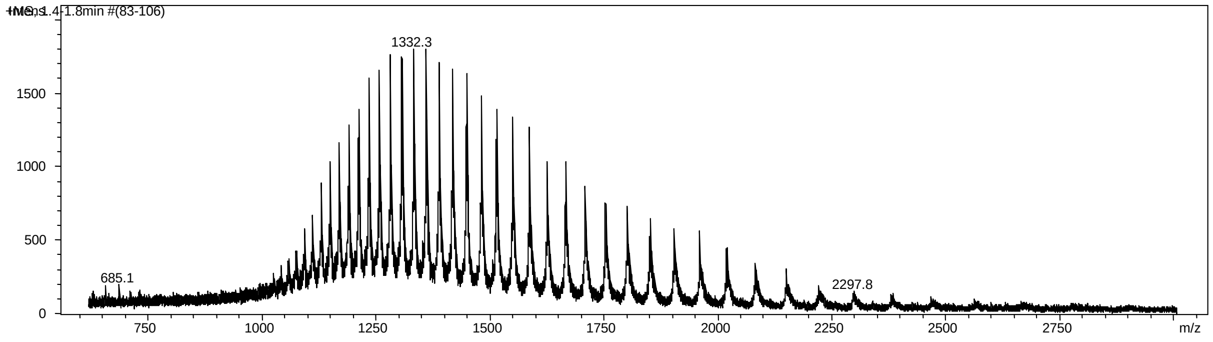

(c)

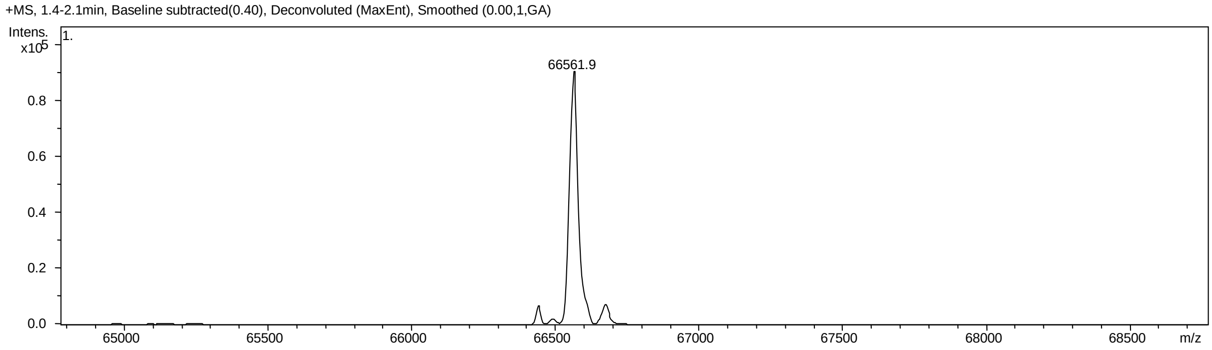

Figure S10. (a) TIC, (b) non-deconvoluted and (c) deconvoluted MS data for attempted thiol exchange from bioconjugate **5** after 42 h.

95 hours

(a)

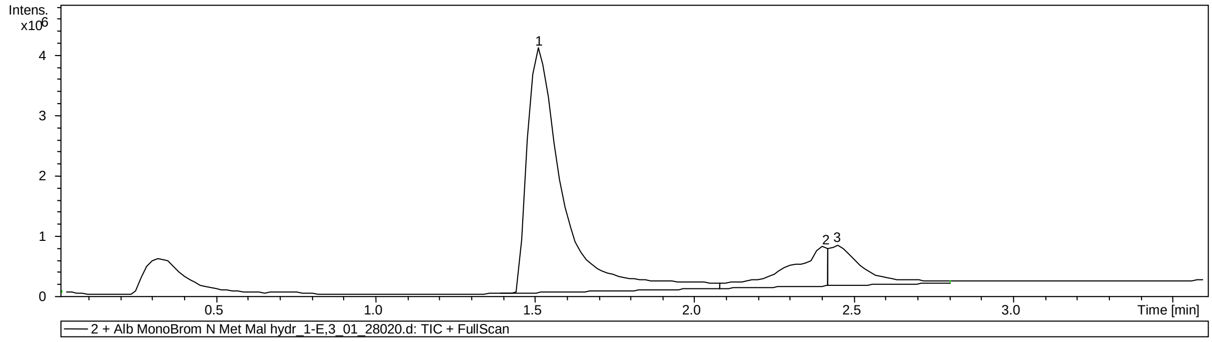

(b)

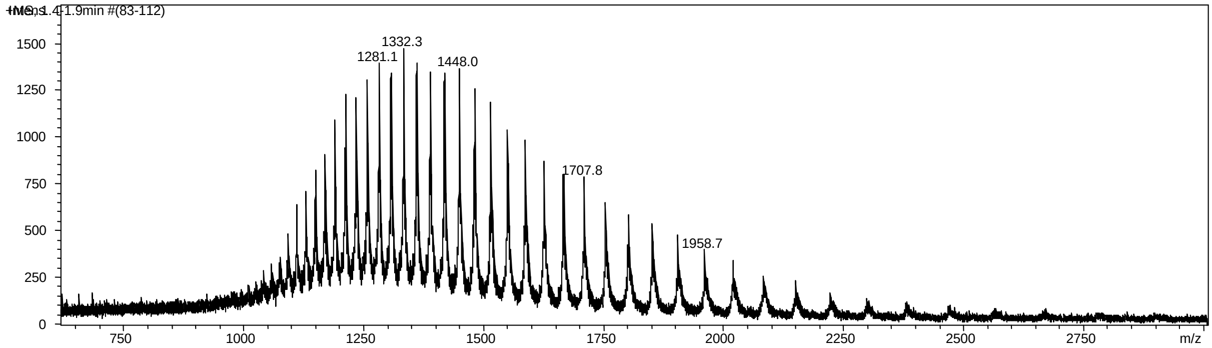

(c)

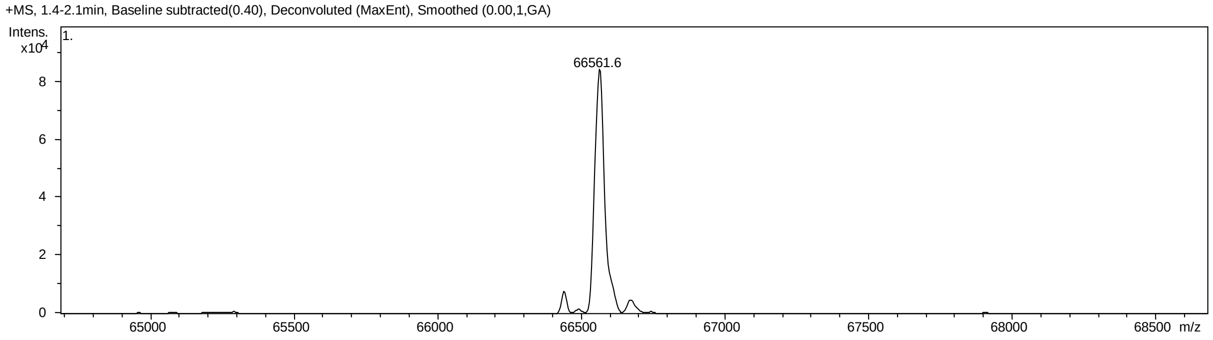

Figure S11. (a) TIC, (b) non-deconvoluted and (c) deconvoluted MS data for attempted thiol exchange from bioconjugate **5** after 95 h.

Stability experiment with bioconjugate 2 in PBS

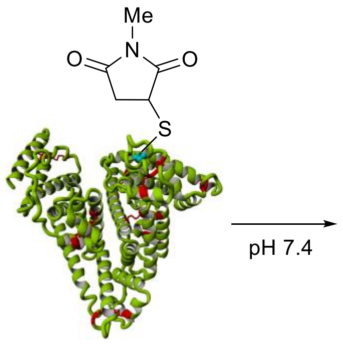

Bioconjugate **2** was diluted with PBS (pH 7.4) to give a final concentration of 19.5 μM. This sample was placed at 37 °C protected from light. The sample was analysed by LC-MS at different times. Expected mass: 66,548 Da (bioconjugate **2**), 66,566 Da (bioconjugate **3**). Observed mass: 66,552 Da at 4 hours, 66,554 Da at 42 hours and 66,558 Da at 95 hours indicating ring hydrolysis to take place without loss of conjugate.

4 hours

(a)

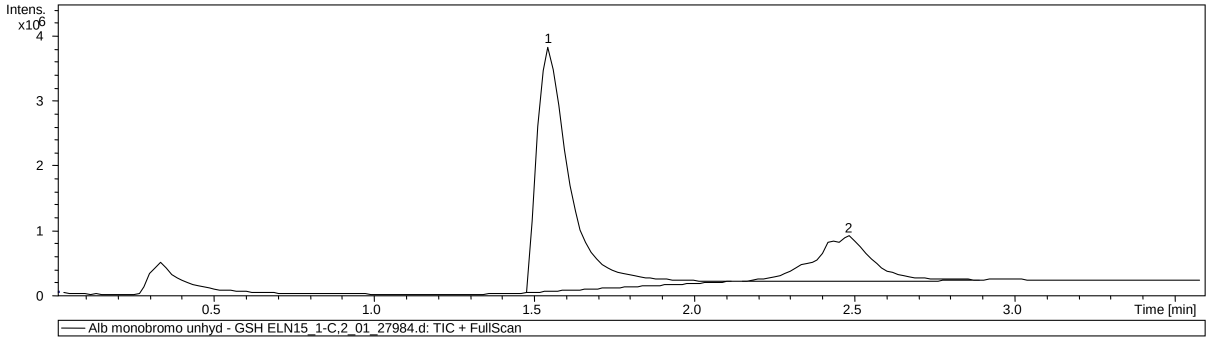

(b)

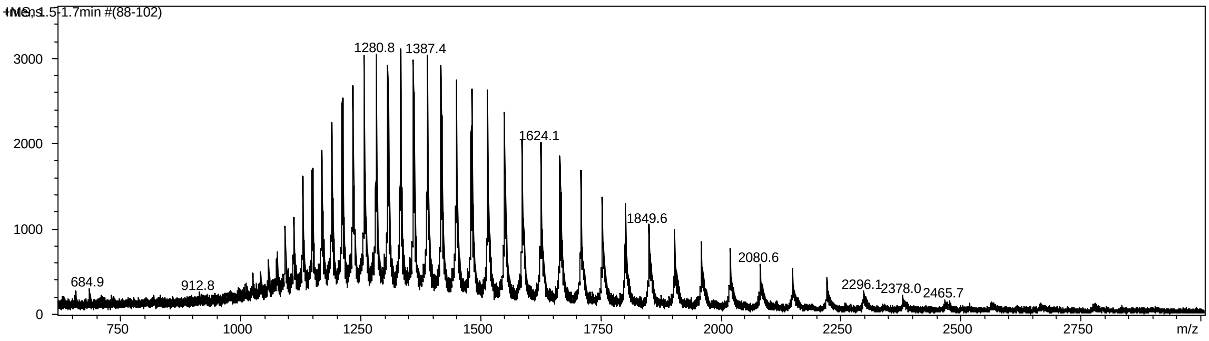

(c)

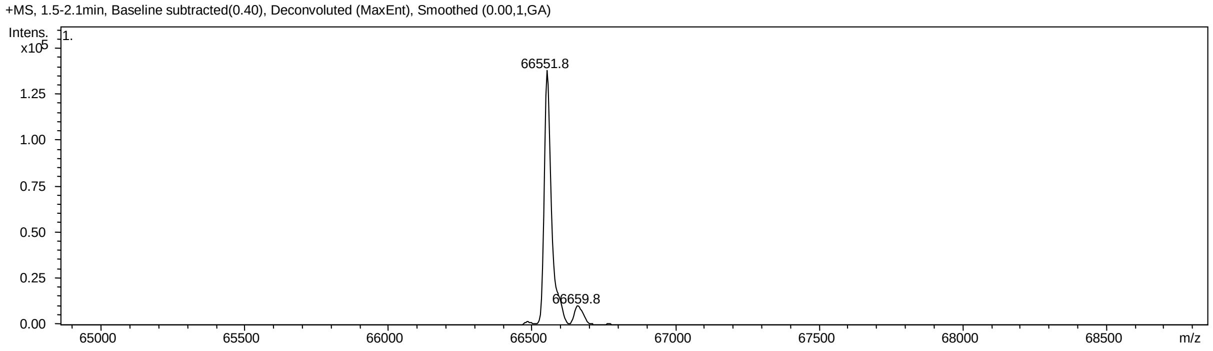

Figure S12. (a) TIC, (b) non-deconvoluted and (c) deconvoluted MS data for stability experiments with bioconjugate **2** after 4 h.

42 hours

(a)

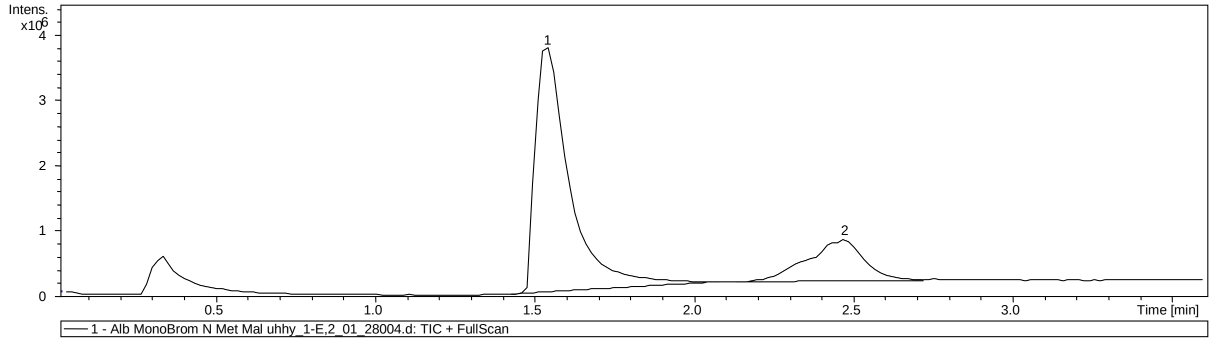

(b)

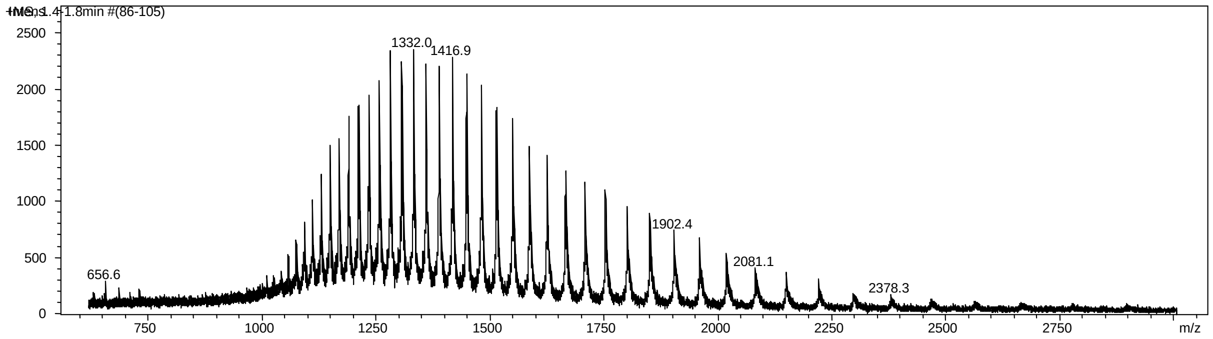

(c)

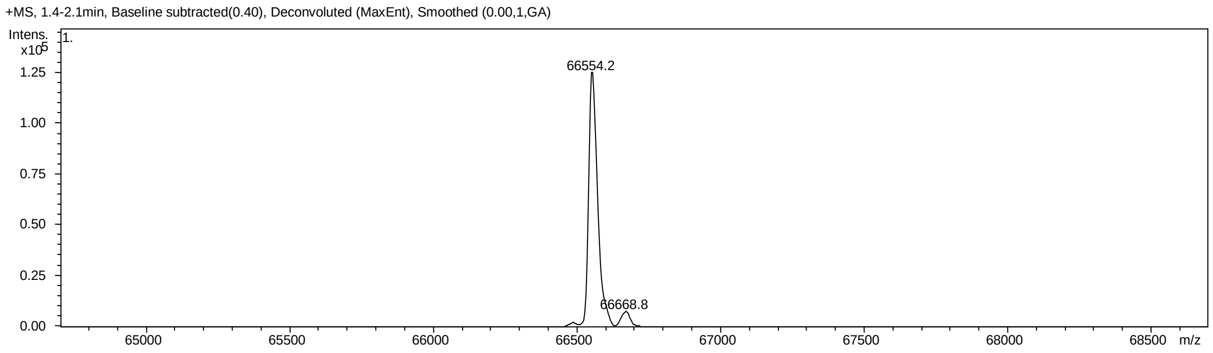

Figure S13. (a) TIC, (b) non-deconvoluted and (c) deconvoluted MS data for stability experiments with bioconjugate **2** after 42 h.

95 hours

(a)

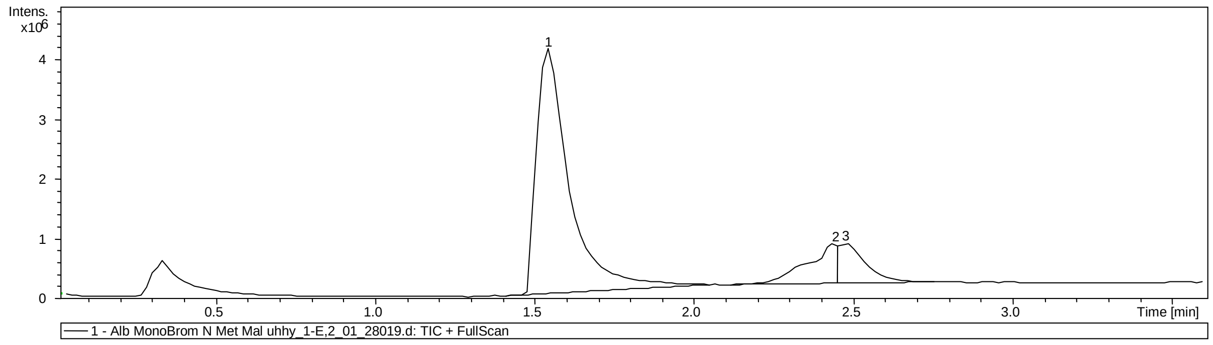

(b)

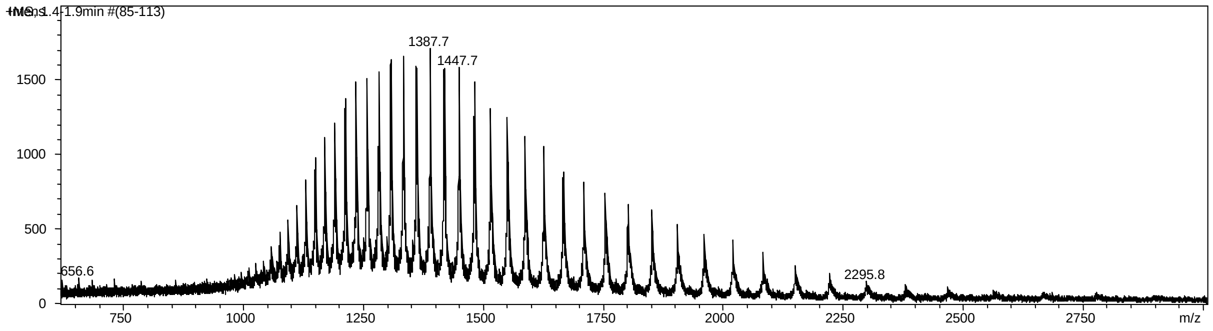

(c)

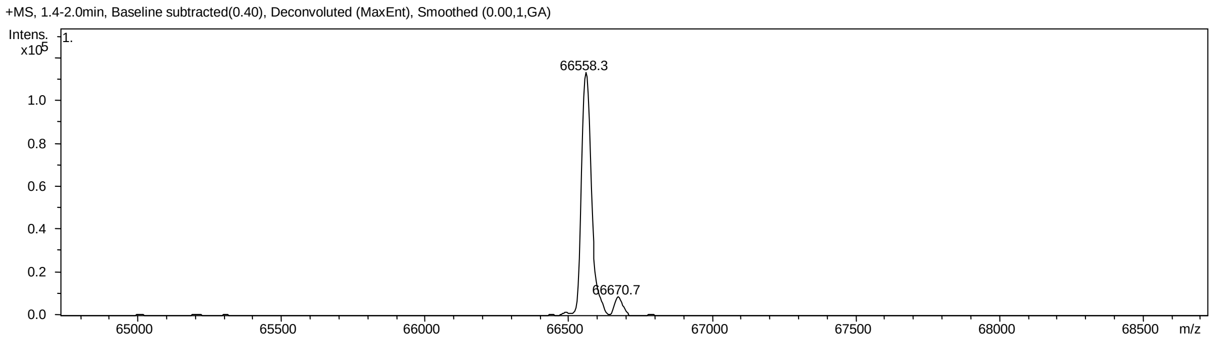

Figure S14. (a) TIC, (b) non-deconvoluted and (c) deconvoluted MS data for stability experiments with bioconjugate **2** after 95 h.

Stability experiment with bioconjugate 2 in PBS with Thiol

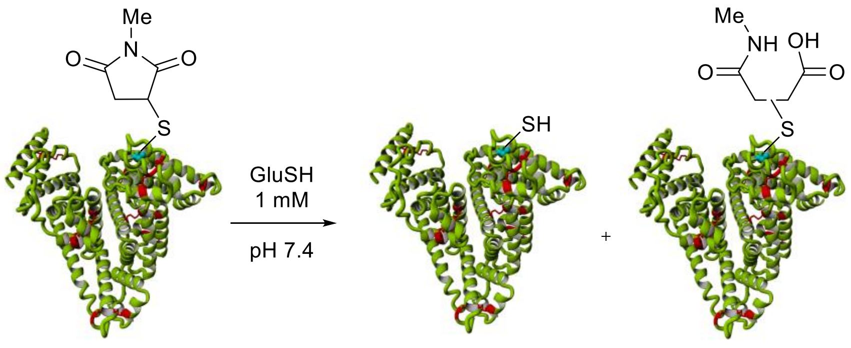

Bioconjugate **2** (53 µL, 37.6 µM, 2.00 nmol) was diluted to a final concentration of 20 µM by adding PBS (pH 7.4) (37 µL) and a solution of GluSH (10 µL, 10 mM, 100 nmol) in PBS (pH 7.4) to give a 50 times excess of GluSH to albumin. This sample was placed at 37 °C protected from light. The sample was analysed by LC-MS at different times. Expected mass: 66,439 Da (albumin **1**), 66,548 Da (bioconjugate **2**), 66,566 Da (bioconjugate **3**), 66,746 Da (albumin with GluSH). Observed mass: 66,440 Da and a small 66,563 Da signals at 4 hours indicating removal of conjugate except from the ring hydrolysed conjugate. At 42 hours main peak at 66441 Da and two minor peaks at 66,563 Da and 66,746 Da. At 95 hours the 66,746 Da peak is apparently further increased at the expense of the 66,440 Da peak with the 66,563 Da remaining unchanged as expected.

4 hours

(a)

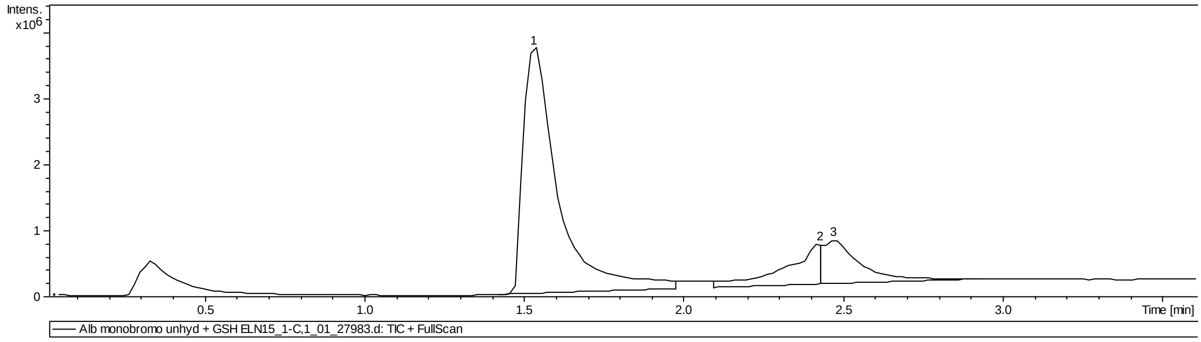

(b)

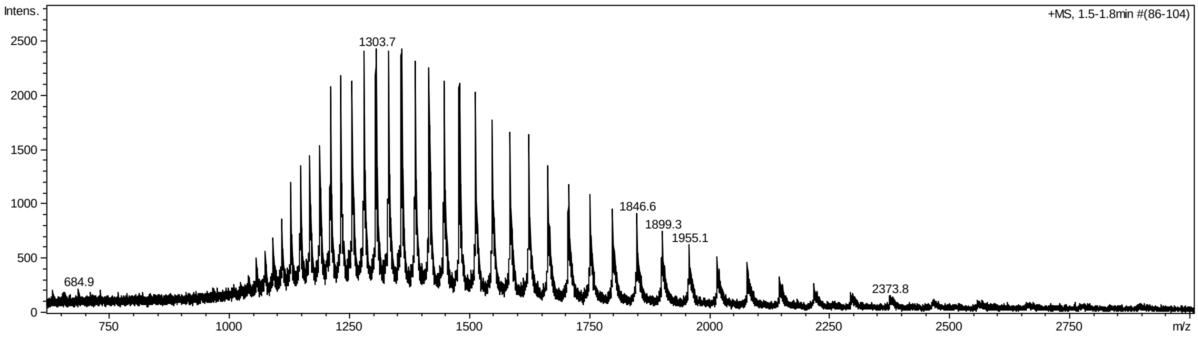

(c)

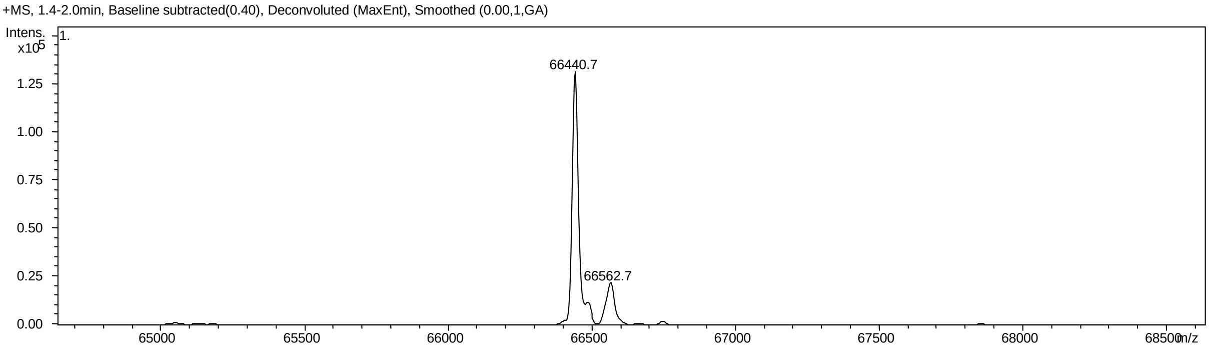

Figure S15. (a) TIC, (b) non-deconvoluted and (c) deconvoluted MS data for attempted thiol exchange from bioconjugate **2** after 4 h.

42 hours

(a)

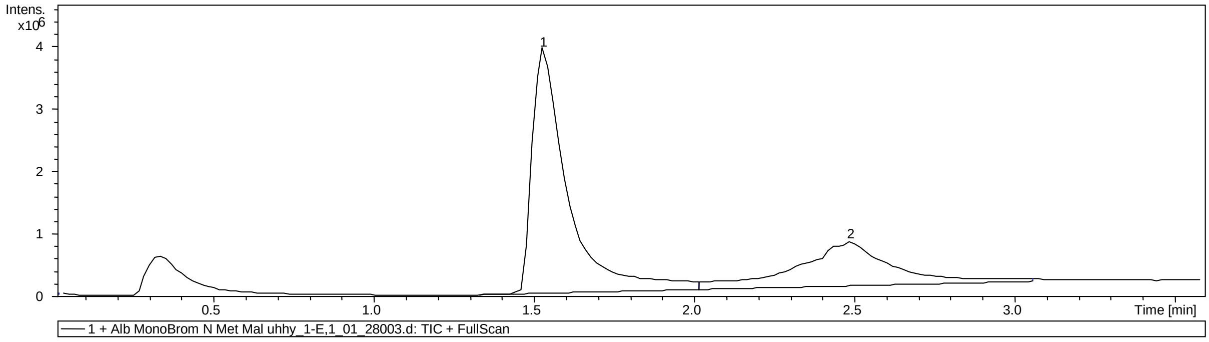

(b)

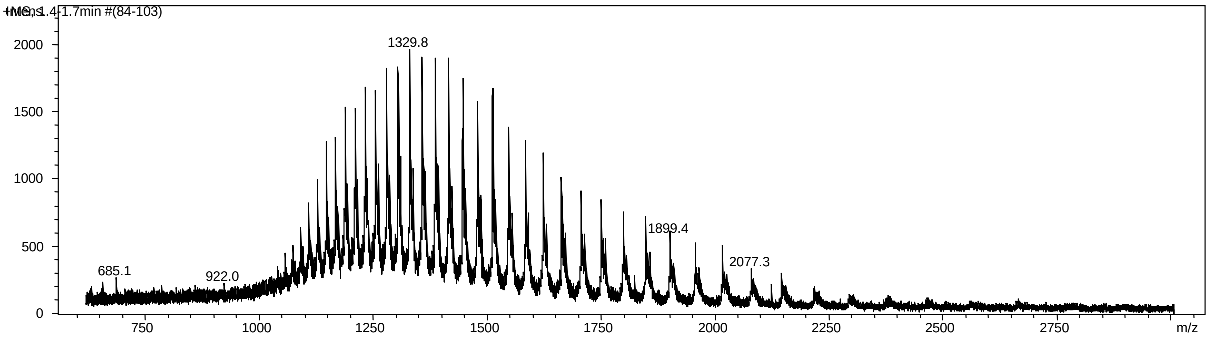

(c)

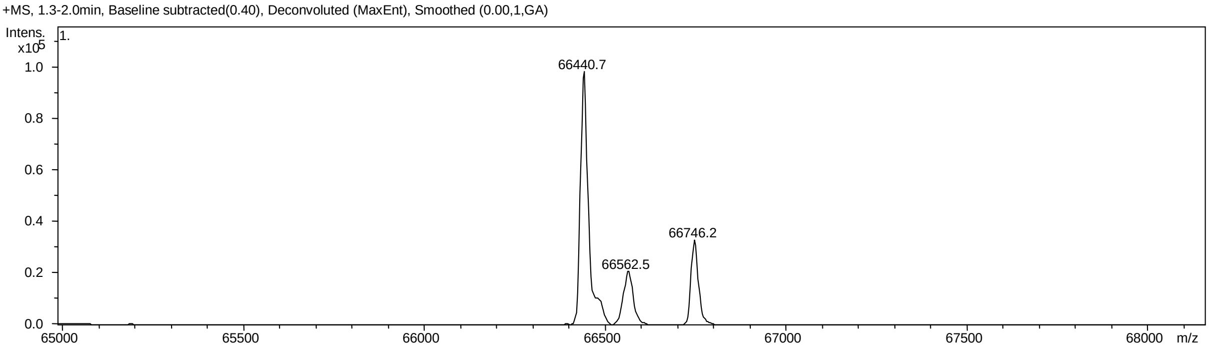

Figure S16. (a) TIC, (b) non-deconvoluted and (c) deconvoluted MS data for attempted thiol exchange from bioconjugate **2** after 42 h.

95 hours

(a)

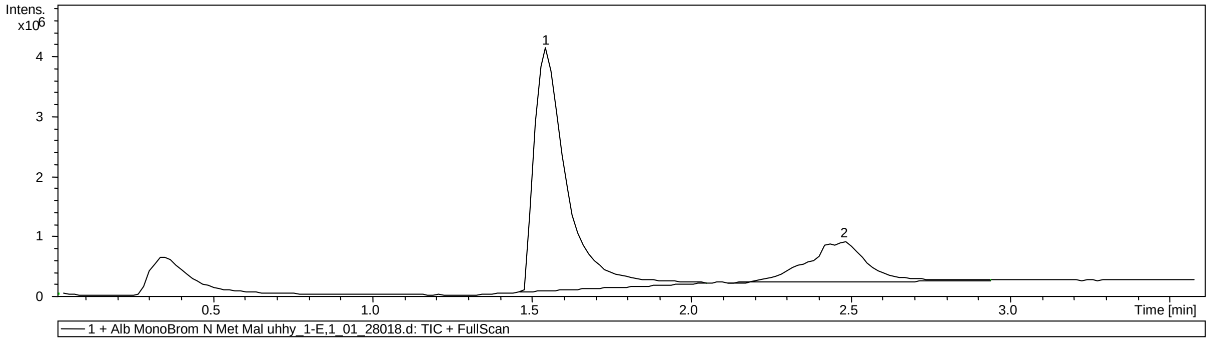

(b)

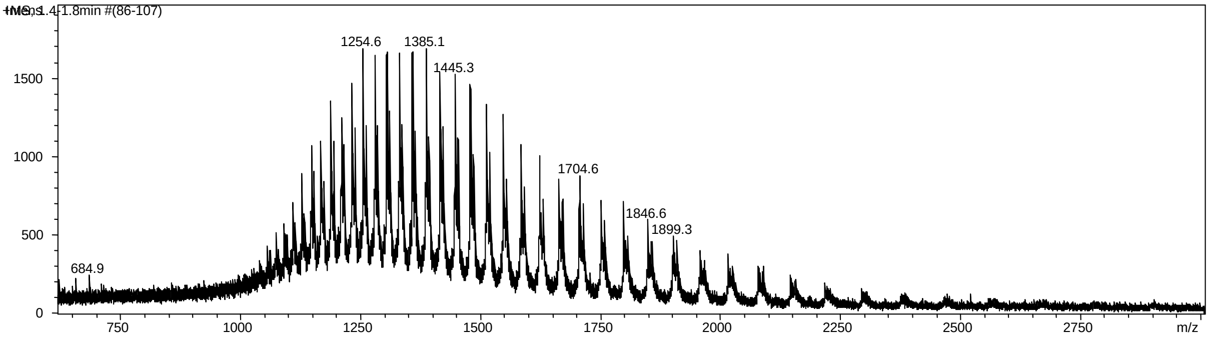

(c)

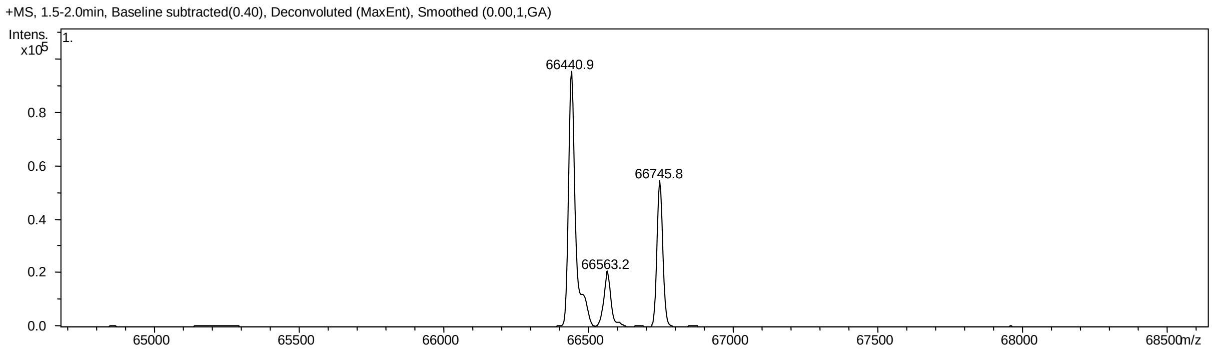

Figure S17. (a) TIC, (b) non-deconvoluted and (c) deconvoluted MS data for attempted thiol exchange from bioconjugate **2** after 95 h.

Reaction of human serum albumin with Alexafluor bromomaleimide

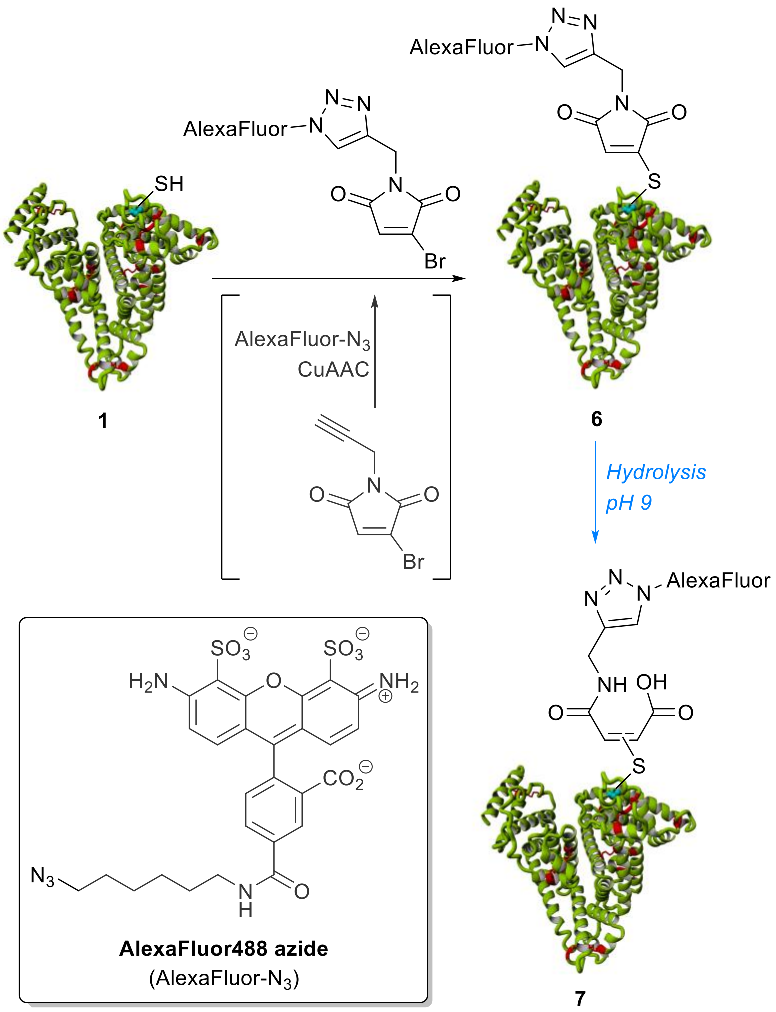

2-(6-Amino-3-iminio-4,5-disulfonato-3,10-dihydroanthracen-9-yl)-5-((5-(4-((3-bromo-2,5-dioxo-2,5-dihydro-1*H*-pyrrol-1-yl)methyl)-1*H*-1,2,3-triazol-1-yl)pentyl) carbamoyl) benzoate (Alexafluro bromomaleimide) was prepared by reaction of 3-bromo-1-(prop-2-yn-1-yl)-1*H*-pyrrole-2,5-dione and Alexa Fluor® 488 azide, conditions as reported by Machida *et al.*[2] using CuBr in place of CuI and THPTA in place of TBTA:[2] THPTA (3.3 µL, 40 mM in dry DMF) was added to a solution of CuBr (3.3 µL, 40 mM in dry acetonitrile). 3-Bromo-1-(prop-2-yn-1-yl)-1*H*-pyrrole-2,5-dione (6.5 µL, 10 mM in dry acetonitrile) was added to the premixed copper solution. Alexa Fluor 488 azide (7.5 µL, 10 mM in dry DMF) was added to the reaction mixture and left under argon overnight at room temperature.

To albumin **1** (50 µL, 100 µM, 5 nmol) in PBS (pH 7.4) was added a freshly prepared solution of 2-(6-amino-3-iminio-4,5-disulfonato-3,10-dihydroanthracen-9-yl)-5-((5-(4-((3-bromo-2,5-dioxo-2,5-dihydro-1H-pyrrol-1-yl)methyl)-1H-1,2,3-triazol-1-yl)pentyl) carbamoyl) benzoate (5 µL, 2.5 mM, 12.5 nmol) in PBS (pH 7.4), and the reaction mixture incubated at room temperature for 1 h. The small molecules were removed by ultrafiltration using a Vivaspin 500 (MWCO 5,000 Generon). Conjugate **6** was characterized by LC-MS and purity checked by SEC-HPLC (220 nm). Expected mass: 67,230 Da (bioconjugate **6**). Observed mass: 67,204 Da. UV-Vis absorption spectrum of the conjugate gave a fluorophore to albumin ratio of 1.03:1.

(a)

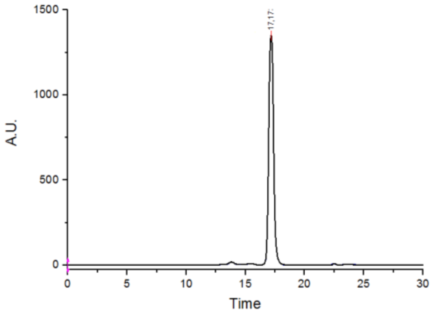
A.U.
Time
17.17

(b)

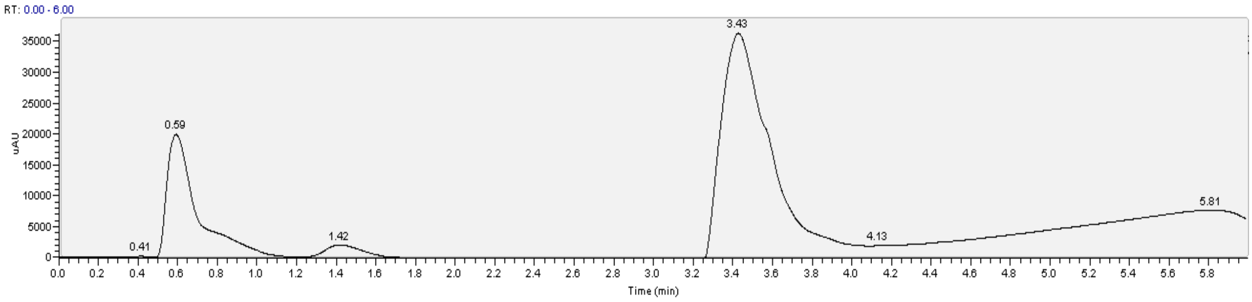
RT: 0.00 - 6.00
uAU
Time (min)
0.41
0.59
1.42
3.43
4.13
5.81

(c)

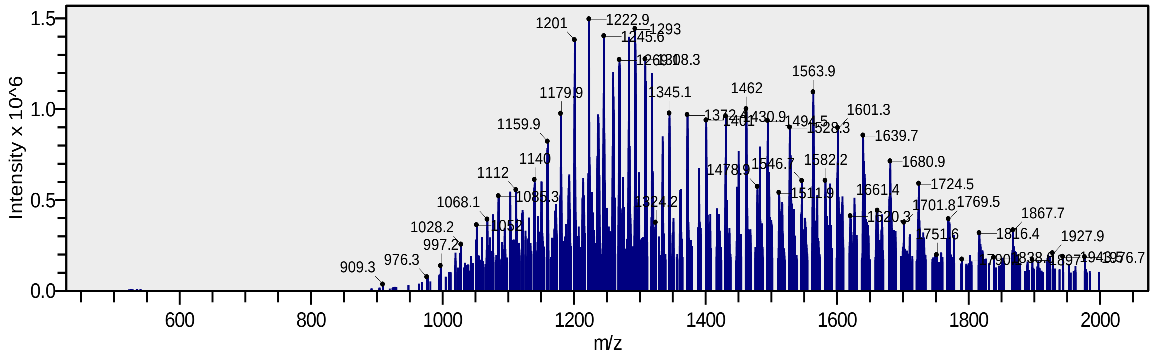
Intensity x 10^6
m/z
1201
1222.9
1293
1245.6
1179.9
1345.1
1462
1563.9
1601.3
1639.7
1680.9
1724.5
1769.5
1867.7
1927.9
909.3
976.3
997.2
1028.2
1068.1
1112
1140
1159.9

(d)

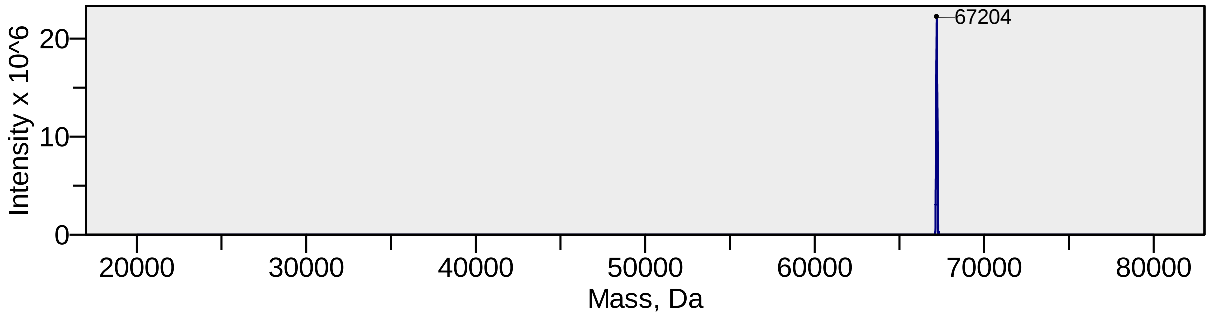
Intensity x 10^6
Mass, Da
67204

(e)

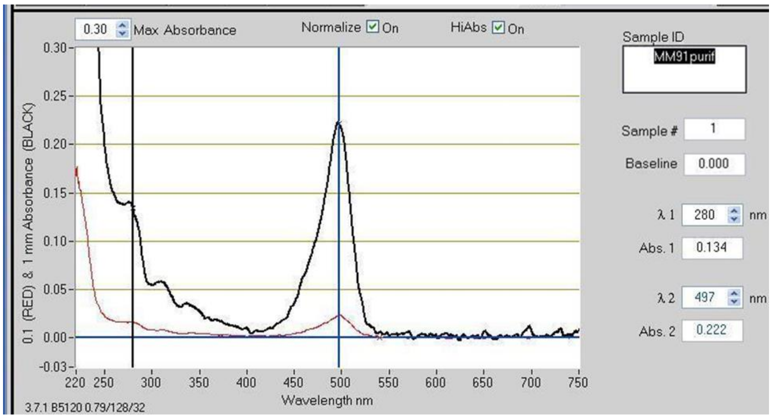

Figure S18. (a) SEC-HPLC chromatogram (λ = 220 nm), (b) TIC (c) non-deconvoluted, (d) deconvoluted MS data for formation of bioconjugate **6**, (e) UV-Vis absorption spectrum of bioconjugate **6**.

To promote hydrolysis of the bridging agent, conjugate **6** was buffer swapped into borate buffer (0.1 M, 1 mM EDTA, pH 9) and incubated for 24 h at 37 °C. An aliquot (5 µL) was diluted four times with water and analysed by LC-MS. Observed masses: 67,221 Da. Expected mass: 67,248 Da. UV-Vis absorption spectrum of the conjugate gave a fluorophore to albumin ratio of 1.02:1.

(a)

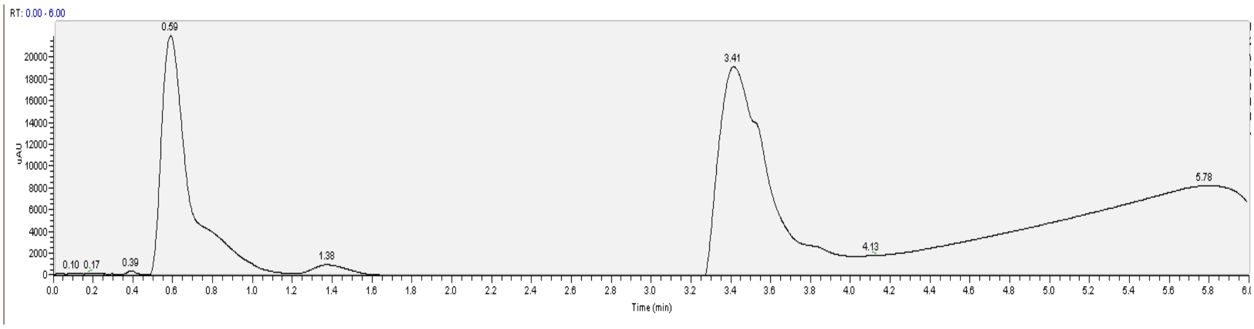

(b)

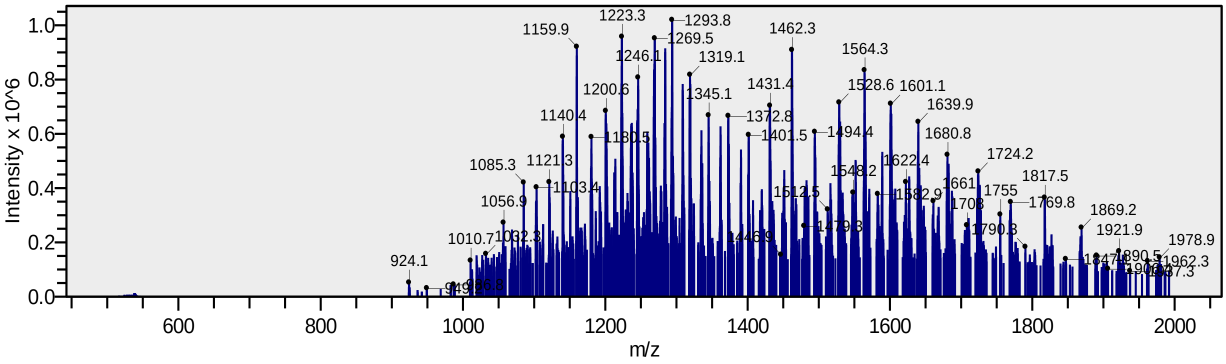

(c)

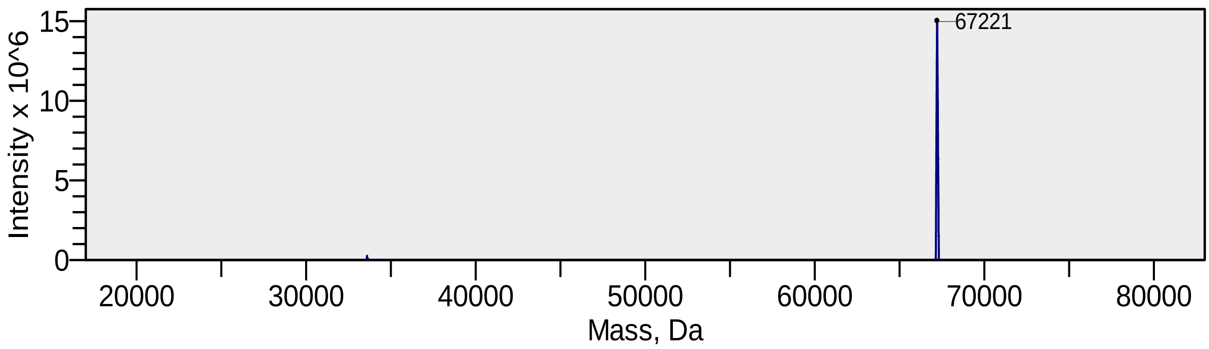

(d)

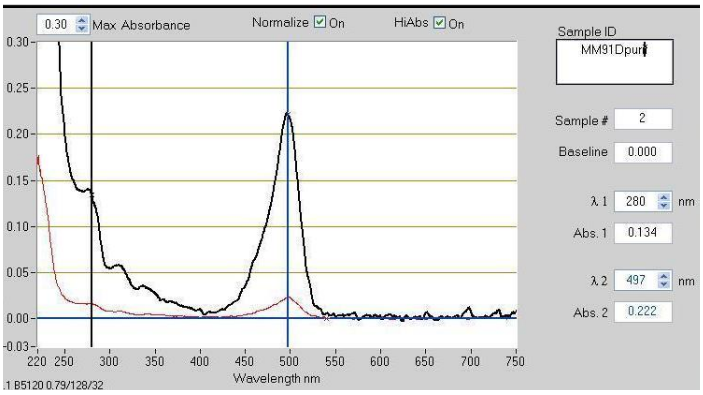

Figure S19. (a) TIC, (b) non-deconvoluted, (c) deconvoluted MS data for formation of bioconjugate **7** and (d) UV-Vis absorption spectrum of bioconjugate **7**.

References

1 M.G. Banwell, M.T. Jones, D.T.J. Long, D.W. Lupton, D.M. Pinkerton, J.K. Ray and A.C. Willis, *Tettrahedron*, 2010, **66**, 9252-9262.

2 T. Machida, K. Lang, L. Xue, J. W. Chin and N. Winssinger, Site-Specific Glycoconjugation of Protein via Bioorthogonal Tetrazine Cycloaddition with a Genetically Encoded trans- Cyclooctene or Bicyclononyne, *Bioconjugate Chem.*, 2015, DOI: 10.1021/acs.bioconjchem.5b00101.
